# The Mitochondrial Genome of the Phytopathogenic Fungus *Bipolaris sorokiniana* and the Utility of Mitochondrial Genome to Infer Phylogeny of Dothideomycetes

**DOI:** 10.3389/fmicb.2020.00863

**Published:** 2020-05-08

**Authors:** Nan Song, Yuehua Geng, Xinghao Li

**Affiliations:** College of Plant Protection, Henan Agricultural University, Zhengzhou, China

**Keywords:** fungi, Ascomycota, *Pleosporales*, next-generation sequencing, mitogenome, phylogenetic

## Abstract

A number of species in *Bipolaris* are important plant pathogens. Due to a limited number of synapomorphic characters, it is difficult to perform species identification and to estimate phylogeny of *Bipolaris* based solely on morphology. In this study, we sequenced the complete mitochondrial genome of *Bipolaris sorokiniana*, and presented the detailed annotation of the genome. The *B. sorokiniana* mitochondrial genome is 137,775 bp long, and contains two ribosomal RNA genes, 12 core protein-coding genes, 38 tRNA genes. In addition, two ribosomal protein genes (*rps3* gene and *rps5* gene) and the fungal mitochondrial RNase P gene (*rnpB*) are identified. The large genome size is mostly determined by the presence of numerous intronic and intergenic regions. A total of 28 introns are inserted in eight core protein-coding genes. Together with the published mitochondrial genome sequences, we conducted a preliminary phylogenetic inference of Dothideomycetes under various datasets and substitution models. The monophyly of *Capnodiales*, *Botryosphaeriales* and *Pleosporales* are consistently supported in all analyses. The *Venturiaceae* forms an independent lineage, with a distant phylogenetic relationship to *Pleosporales*. At the family level, the *Mycosphaerellaceae*, *Botryosphaeriaceae*. *Phaeosphaeriaceae*, and *Pleosporaceae* are recognized in the majority of trees.

## Introduction

The genus *Bipolaris* belongs to the family *Pleosporaceae* (Ascomycota, Dothideomycetes, *Pleosporales*). Shoemaker (1959) originally established the generic *Bipolaris*. The species of *Bipolaris* were once classified in *Helminthosporium* (Link, 1824). Subramanian (1971) provided a key to separate the species of *Bipolaris* from members of the *Helminthosporium*. Further study divided the *Helminthosporium* into several genera including *Bipolaris*, *Curvularia*, *Drechslera*, and *Exserohilum* (Sivanesan, 1987). Modern descriptions and illustrations for the species in the genus *Bipolaris* were given in the studies of Manamgoda et al. (2012, 2014). Many morphological similarities are shared by *Bipolaris* and *Curvularia*, both of which have sexual morphs in the genus *Cochliobolus* (Alcorn, 1983; Manamgoda et al., 2014). The genera *Cochliobolus*, *Bipolaris* and *Curvularia* form a complex, and the taxonomy of this complex are uncertain with the morphological characters (Manamgoda et al., 2012). A combined analysis of ITS, GPDH, EF1-α and LSU gene sequences resulted in two main groups corresponding to the complex (Manamgoda et al., 2012). One clade included the majority of *Bipolaris* and *Cochliobolus*, and another contained the *Curvularia* and the partial members of *Bipolaris* and *Cochliobolus* (Manamgoda et al., 2012). Manamgoda et al. (2014) recovered *Bipolaris* as a sister group to *Curvularia* based on a combined analysis of ITS, GPDH and EF1-α gene sequences.

*Bipolaris sorokiniana* (Sacc.) Shoemaker [teleomorph: *Cochliobolus sativus* (Ito and Kuribayashi) Drechs. ex Dastur] is a seed and soil borne pathogen, which causes spot blotch, root rot, leaf spot, seedling blight, head blight, and black point disease in cereal crops (Wiese, 1998; Kumar et al., 2002). Among these diseases, the leaf spot blotch is recognized as the major biotic stress hampering commercial production of wheat, because it results in significant yield losses (Chowdhury et al., 2013; Mina et al., 2016). The type strain was isolated from Russia with number of MBT197973 in 1890, which was originally named as *Helminthosporium sorokinianum*. Several synonyms of *B. sorokiniana* were used as following: *H. sorokinianum*, *H. sativum*, *Drechslera sorokiniana* (Maraite et al., 1998). The detailed description of *B. sorokiniana* can be seen from Commonwealth Mycological Institute’s Sivanesan and Holliday (1981). In addition to *B. sorokiniana*, the genus *Bipolaris* contains many other important plant pathogens with worldwide distribution. The distinction of species in *Bipolaris* and the classification of *Bipolaris* with other fungus species appear difficult when only morphological characters are considered. Because some species have overlapping morphological features. Molecular phylogenetic studies based on gene sequences have shown promise in resolving the fungi classification problems (Swann and Taylor, 1995; Berbee et al., 1999; Fitzpatrick et al., 2006; James et al., 2006a, b; Robbertse et al., 2006; Mangold et al., 2008; Wang et al., 2009; Manamgoda et al., 2012, 2014). Ohm et al. (2012) determined 14 dothideomycete whole-genomes, including that from the sexual morph of *B. sorokiniana* (i.e., *Cochliobolus sativus*). Condon et al. (2013) reported three additional whole-genome sequences of *Bipolaris*, namely the *B. victoriae* (as *C. victoriae*), *B. zeicola* (as *C. carbonum*), and *B. maydis* (as *C. heterostrophus*).

Mitochondrial (mt) DNA has been widely used in evolutionary biology and systematics of Fungi (Paquin et al., 1997; Hausner, 2003; Kouvelis et al., 2004; Formighieri et al., 2008; Pantou et al., 2008; Sethuraman et al., 2009; Duo et al., 2012; Aguileta et al., 2014; Lin et al., 2015; Utomo et al., 2019). As a class of molecular markers, mtDNA sequences have some preferable characteristics in resolving systematic issues, such as faster rate of evolution, the virtual absence of recombination, and conserved gene content (Simon et al., 1994, 2006; Caterino et al., 2000; Lin and Danforth, 2004). Recently, development of high-throughput sequencing technologies and related bioinformatics tools have allowed considerably greater numbers of nucleotides to be characterized and the genome-scale data to be more easily assembled. As a case in point, next-generation sequencing (NGS) technologies have been successfully used to reconstruct the fungal mitochondrial genomes (Losada et al., 2014; Mardanov et al., 2014; Salavirta et al., 2014; Torriani et al., 2014; Lin et al., 2015; Kanzi et al., 2016; Nowrousian, 2016; Kang et al., 2017; Deng et al., 2018). Compared to the whole-genome data, mitochondrial genomes are more readily sequenced with broader taxon sampling and a reasonable cost. The fungal mitochondrial genome size varied greatly between species. For example, the mitochondrial genome of *Hanseniaspora uvarum* has a length of 18,844 bp (Pramateftaki et al., 2006), while the *Sclerotinia borealis* has a length of 203,051 bp (Mardanov et al., 2014). Large variation of mitochondrial genome size in fungi is mainly attributed to different lengths of intronic sequences and intergenic regions (Belcour et al., 1997; Hausner, 2003; Formighieri et al., 2008). Despite with the continuously increasing number of fungal mitochondrial genome studies (Paquin et al., 1997; Hausner, 2003; Formighieri et al., 2008; Pantou et al., 2008; Duo et al., 2012; Torriani et al., 2014; Kang et al., 2017; Zaccaron and Bluhm, 2017; Deng et al., 2018; Utomo et al., 2019), the complete mtDNA sequences available for some important lineages are still limited. There are only 16 complete mitochondrial genomes published from the class Dothideomycetes in GenBank as of December 2019. The *Bipolaris cookei* was the first species having complete mtDNA sequence (Zaccaron and Bluhm, 2017) in the genus *Bipolaris*. It provided a reference for further investigation of the *Bipolaris* mitochondrial genomes.

In the present study, we use an NGS based approach to determine a complete mitochondrial genome of *B. sorokiniana*. The description of the mitochondrial genome organization is presented. One of our primary objectives is to explore the utility of mtDNA sequence data in inferring phylogeny of Dothideomycetes.

## Materials and Methods

### Fungal Isolate

The strains of *B. sorokiniana* were obtained from wheat root and leaves in Luyi County (38.48°N, 115.08°E), Henan province, China, with the single spore isolation (Matusinsky et al., 2010). The living strains were harvested in 10% aqueous glycerol in a 2 ml-Eppendorf tube, and then were stored at −80°C. The fungus was cultured on PDA culture medium at 25°C, with ampicillin resistant to bacterial growth. Species identification was based on morphological features and confirmed using ITS sequence. The dried cultures and living cultures of specimen have been deposited in the Herbarium of Henan Agricultural University: Fungi (HHAUF).

### DNA Extraction and Genome Sequencing

Total genomic DNA was extracted by using the Cetyl Trimethyl Ammonium Bromide (CTAB) method (Rogers and Bendich, 1985), with minor modification. DNA concentration (avg. 14.20 ng/μL) was determined using a Qubit Flurometer (Invitrogen, United States) and a NanoDrop Spectrophotometer (Thermo Scientific, United States).

Genomic DNA were sent to Shanghai Personal Biotechnology Co., Ltd. for library preparation and high-throughput sequencing. Library was constructed by using the Illumina TruSeqTM DNA Sample Prep Kit (Illumina, San Diego, CA, United States), with the insert size of 400 bp. The genome sequencing was conducted on an Illumina NovaSeq 6000 platform, with a strategy of 150 paired-end sequencing.

### Genome Assembly and Annotation

Raw data set was filtered through AdapterRemoval (Lindgreen, 2012) and SOAPec_v2.01 (Luo et al., 2012). The high-quality reads (Q20 = 97.71%, and Q30 = 93.43%) were used to assemble the mitochondrial contig, with the software MITObim v1.9 (Hahn et al., 2013). The mitochondrial genome of *B. cookei* (Zaccaron and Bluhm, 2017) was used as the reference genome. We also used IDBA-tran v. 1.1.1 (Peng et al., 2013) to conduct *de novo* assembly of *B. sorokiniana*, with the following parameters: the minimum size of contig of 200, an initial k-mer size of 41, an iteration size of 10, and a maximum k-mer size of 91. The assembled result produced by IDBA-tran were used to build a Blast database, using the program makeblastdb implemented in the BLAST package v 2.9.0+ (Camacho et al., 2009). The mitochondrial gene sequences of *B. cookei* were used as queries in Blastn searches against the database. BLAST hit contigs were retrieved through the program blastdbcmd in BLAST package v 2.9.0+ (Camacho et al., 2009).

To check the quality of assembly, we used BWA v. 0.7.5 (Li and Durbin, 2009) to align the sequenced reads to the mtDNA sequence assembled. SAMtools v. 0.1.19 (Li et al., 2009) was used to convert SAM output to a sorted BAM file. Qualimap v. 2.2.1 (Okonechnikov et al., 2016) was applied to calculate correctly mapped reads to the mitochondrial genome.

**FIGURE 1:**
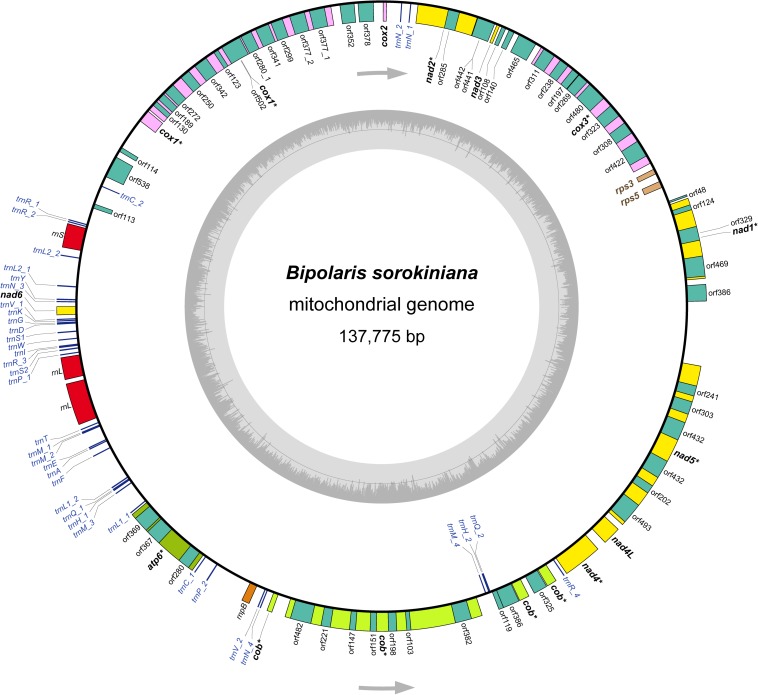
The structure of the mitochondrial genome of *Bipolaris sorokiniana*. Arrows indicate the direction of gene transcription. The inner circles show the GC content. All genes identified are indicated in italics. The core 12 protein-coding genes are highlighted in bold. Of which, eight protein-coding genes including introns are denoted by asterisk. The detailed annotation of the mitochondrial genome of *B. sorokiniana* are shown in Supplementary Table S2.

The mitochondrial genome annotation was conducted in MITOS web (Bernt et al., 2013). The following settings were implemented: Reference, “RefSeq 63 Fungi”; Genetic Code, “4 Mold.” We also used the automated organelle genome annotation tool MFannot (Valach et al., 2014) to perform annotation of the mitochondrial genome of *B. sorokiniana*, under the genetic code of “4 Mold, Protozoan, and Coelenterate Mitochondrial; Mycoplasma/Spiroplasma.” The gene boundaries were further refined by sequence alignment to the *B. cookei* mitochondrial genome (Zaccaron and Bluhm, 2017). The secondary structures of tRNA genes were predicted by MITOS (Bernt et al., 2013), and redrawn in Adobe Illustrator CS6. The genome structure image (Figure 1) was generated using OGDRAW (Greiner et al., 2019). The newly determined mitochondrial genome sequence of *B. sorokiniana* has been submitted to GenBank under the accession number MN978926. Besides the *B. sorokiniana* mitochondrial genome sequenced in this study, all other 19 complete mtDNA sequences (sixteen representing the Dothideomycetes and three representing the Sordariomycetes, see details in Table 1) downloaded from GenBank were re-annotated in MITOS web (Bernt et al., 2013) following the settings described above for uniform annotations.

**TABLE 1:** Species included in the phylogenetic analyses.

Item	Phylum	Class	Order	Family	Species	Accession number
Outgroup	Ascomycota	Sordariomycetes	*Hypocreales*	*Nectriaceae*	*Fusarium culmorum*	KP827647
	Ascomycota	Sordariomycetes	*Hypocreales*	*Nectriaceae*	*Fusarium circinatum*	JX910419
	Ascomycota	Sordariomycetes	*Hypocreales*	*Nectriaceae*	*Fusarium oxysporum*	KR952337
Ingroup	Ascomycota	Dothideomycetes	*Venturiales*	*Venturiaceae*	*Venturia effusa*	CP042205
	Ascomycota	Dothideomycetes	*Capnodiales*	*Mycosphaerellaceae*	*Zymoseptoria tritici*	MH374028
	Ascomycota	Dothideomycetes	*Capnodiales*	*Mycosphaerellaceae*	*Zasmidium cellare*	NC_030334
	Ascomycota	Dothideomycetes	*Capnodiales*	*Mycosphaerellaceae*	*Pseudocercospora mori*	NC_037198
	Ascomycota	Dothideomycetes	*Capnodiales*	*Mycosphaerellaceae*	*Pseudocercospora fijiensis*	NC_044132
	Ascomycota	Dothideomycetes	*Botryosphaeriales*	*Botryosphaeriaceae*	*Botryosphaeria dothidea*	KY801668
	Ascomycota	Dothideomycetes	*Botryosphaeriales*	*Botryosphaeriaceae*	*Botryosphaeria kuwatsukai*	MG593780
	Ascomycota	Dothideomycetes	*Pleosporales*	*Astrosphaeriellaceae*	*Pithomyces chartarum*	NC_035636
	Ascomycota	Dothideomycetes	*Pleosporales*	*Didymellaceae*	*Didymella pinodes*	NC_029396
	Ascomycota	Dothideomycetes	*Pleosporales*	*Phaeosphaeriaceae*	*Phaeosphaeria nodorum*	CM017955
	Ascomycota	Dothideomycetes	*Pleosporales*	*Phaeosphaeriaceae*	*Shiraia bambusicola*	NC_026869
	Ascomycota	Dothideomycetes	*Pleosporales*	*Coniothyriaceae*	*Coniothyrium glycines*	NC_040008
	Ascomycota	Dothideomycetes	*Pleosporales*	*Pleosporaceae*	*Pyrenophora teres*	CM017819
	Ascomycota	Dothideomycetes	*Pleosporales*	*Pleosporaceae*	*Alternaria alternata*	MF669499
	Ascomycota	Dothideomycetes	*Pleosporales*	*Pleosporaceae*	*Stemphylium lycopersici*	NC_036039
	Ascomycota	Dothideomycetes	*Pleosporales*	*Pleosporaceae*	*Bipolaris sorokiniana*	MN978926
	Ascomycota	Dothideomycetes	*Pleosporales*	*Pleosporaceae*	*Bipolaris cookei*	MF784482

### Composition of Mitochondrial Genome

The nucleotide composition of the mtDNA sequence of *B. sorokiniana* and other 16 dothideomycete fungi downloaded from GenBank were computed with MEGA 7 (Kumar et al., 2016). The AT and GC skews were calculated according to the formula AT skew = (A - T)/(A + T) and GC skew = (G - C)/(G + C) (Perna and Kocher, 1995), in order to measure the strand-specific bias of nucleotide composition. Moreover, codon usage in the core mitochondrial protein-encoding genes were analyzed in MEGA 7 (Kumar et al., 2016).

### Sequence Alignments

Prior to aligning each mitochondrial gene, we merged the FASTA files generated by MITOS for all species into a single file. Using the custom Perl script (selectSeqs.pl, commands used are provided in Supplementary File S1), the annotated genes collected from each species were extracted to compile the matrices. For the core protein-coding gene matrix, we firstly used TranslatorX (Abascal et al., 2010) to construct a preliminary multiple sequence alignment. The following parameters were set: Genetic code = “Mold mitochondrial,” and Protein alignment = “MAFFT.” Because many protein-coding genes were split into fragments by introns, the initial alignment contained many long gap sequences. In order to remove these gap sequences, we divided an alignment into several shorter ones based on the sequence homology checked by eye. After removal of long gap sequences, the alignments were concatenated into a single matrix, with the Perl script FASconCAT_v1.0 (Kuck and Meusemann, 2010). Ambiguous positions in the genes’ alignment were trimmed with Gblocks (Talavera and Castresana, 2007), under less stringent options. Stop codons in each alignment were checked in MEGA 7 (Kumar et al., 2016) and deleted. Individual gene alignments were concatenated into an alignment comprising all 13 core protein-coding genes but *atp8*. Because this gene is missing in nine out of 20 fungal mitochondrial genomes analyzed. The mitochondrial rRNA and tRNA genes were individually aligned using MAFFT online server with E-INS-i strategy (Katoh and Standley, 2013). For the tRNA gene with more than one copy, only the one most similar to other species was used in the sequence alignment. Poorly aligned sections were eliminated by Gblocks (Talavera and Castresana, 2007). The individual alignments were concatenated to create the data matrices of rRNA gene (rrn) and tRNA gene (trn), respectively.

### Characteristics of Sequence Alignments

Sequence potential saturation was assessed in DAMBE software (Xia, 2013). Sequence divergence heterogeneity was evaluated using AliGROOVE (Kuck et al., 2014), with the default sliding window size. Indels in nucleotide data set were treated as ambiguity and a BLOSUM62 matrix was used as default amino acid substitution matrix. The number of synonymous substitutions per synonymous site (*Ks*) and the number of non-synonymous substitutions per non-synonymous site (*Ka*) for protein-coding genes were estimated with DnaSP v5 (Librado and Rozas, 2009), under the genetic code of mtDNA Mold-Protozoan. Congruence between different gene type alignments (protein-coding genes, rRNA genes, and tRNA genes) was assessed by using the Incongruence Length Difference (ILD) tests (Farris et al., 1994) implemented in PAUP^∗^4.0b10 (Swofford, 2002), under the parsimony optimality criterion and using 1,000 additional replicates.

### Phylogenetic Reconstructions

In the phylogenetic analyses, our taxon sample included 17 fungus species representing eight families of Dothideomycetes, namely *Venturiaceae*, *Mycosphaerellaceae*, *Botryosphaeriaceae*, *Astrosphaeriellaceae*, *Didymellaceae*, *Phaeosphaeriaceae*, *Coniothyriaceae*, and *Pleosporaceae* (Table 1). In addition, three mitochondrial genome sequences from the class Sordariomycetes were selected as outgroups (Fourie et al., 2013; Kulik et al., 2016).

A total of five concatenated datasets were compiled as following: (1) PCG_nt (10,561 nucleotide sites), nucleotide alignment including 12 protein-coding genes; (2) PCG_aa (3,364 amino acid sites), amino acid alignment including 12 protein-coding genes; (3) PCG-rrn (14,612 nucleotide sites), nucleotide alignment including 12 protein-coding genes and two rRNA genes; (4) rrn (4,051 nucleotide sites), nucleotide alignment including *rrnL* and *rrnS* genes; and (5) trn (1,637 nucleotide sites), nucleotide alignment including 22 tRNA genes. The sequence alignments supporting the phylogenetic results of this article are presented in Supplementary File S2.

Phylogenetic trees were built using maximum likelihood (ML) method and Bayesian inference (BI) method. For the combined datasets of PCG_nt, PCG_aa, and PCG-rrn, data partition schemes and best-fitting substitution models (Supplementary Table S1) were estimated using PartitionFinder 2 (Lanfear et al., 2012). Data blocks were predefined by genes. The PartitionFinder 2 analyses were run using a greedy search scheme (Lanfear et al., 2012), with all models considered under the Akaike information criteria.

For ML inferences, we conducted separate analyses of each protein-coding gene (i.e., *atp6*, *cob*, *cox1-3*, and *nad1-6*), and then performed combined analysis based on the dataset of PCG_nt. In addition, other four concatenated datasets (i.e., PCG_aa, PCG-rrn, rrn, and trn) were also used in the ML analyses. ML tree searches were performed in IQ-TREE 1.6.10 (Nguyen et al., 2015) implemented in the Cipres Science Gateway (Miller et al., 2010). Partitioned analyses were conducted for datasets of PCG_nt, PCG_aa, and PCG-rrn, with the data partitions and the best-fitting models selected by PartitionFinder 2 (Lanfear et al., 2012). Allowing partitions to have different speeds (-spp) was selected. Nodal support values (BP) were evaluated through an ultrafast bootstrap approach (Minh et al., 2013), with 10,000 replicates.

To circumvent the long computational time required, only the five concatenated datasets (i.e., PCG_nt, PCG_aa, PCG-rrn, rrn, and trn) were used in Bayesian inferences. Bayesian tree searches were conducted in MrBayes 3.2.6 (Ronquist et al., 2012) implemented in the CIPRES Science Gateway (Miller et al., 2010). We applied the MrBayes blocks for partition definitions generated from PartitionFinder 2 (Lanfear et al., 2012). All model parameters were set as unlinked across partitions. Each analysis involved two independent runs and started from random topology. Each run implemented four Markov chain Monte Carlo chains in parallel for 10 million generations, and sampled every 1,000 generations. The program Tracer 1.7 (Rambaut et al., 2018) was used to analyze the trace files from two Bayesian MCMC runs for monitoring convergence. The first 25% of sampled trees were discarded as burn-in, and the remaining trees were used to calculate a majority-rule consensus tree. Branch support was assessed by clade posterior probabilities (PP).

We also used PhyloBayes (Lartillot et al., 2009) to conduct Bayesian inferences. This software implements the site-heterogeneous CAT-GTR or CAT model accounting for the heterogeneity present in the data. Each analysis involved two independent runs, and started from random topology, respectively. Each run implemented two Markov chain Monte Carlo chains in parallel for at least 20,000 iterations. The CAT-GTR model was used for nucleotide dataset analyses, while the CAT-MTZOA model for amino acid dataset. The “bpcomp” program contained in the package of PhyloBayes was used to calculate the largest (maxdiff) and mean (meandiff) discrepancy observed across all bipartitions. The program “tracecomp” was also used to summarize the discrepancies and the effective sizes estimated for each column of the trace file. When the maxdiff was <0.1 and minimum effective size was >100, the Bayesian runs were recognized to be reached good convergence. The first 1,000 trees of each MCMC were treated as the burn-in, and the majority-rule consensus tree was calculated from the saved trees.

### Hypothesis Testing

We tested the statistical robustness of critical nodes defining deep level relationships in Dothideomycetes, by means of the four-cluster likelihood-mapping (FcLM) approach implemented in IQ-TREE 1.6.10 (Nguyen et al., 2015). The datasets of PCG_nt, PCG_aa, PCG-rrn, rrn, and trn were used for this test. The partition schemes and the corresponding best-fitting models were applied as those in the ML tree searches.

## Results

### Genome Assembly

The total number of raw reads are 22,819,080. After filtering, 22,111,146 high-quality PE reads are produced. A 137,775 bp mitochondrial contig is reconstructed by the assembler MITObim. Statistical analysis from Qualimap show that 465,656 single-mate reads are mapped on this mitochondrial contig. This accounts for 4.2% of the total number of reads. The mean coverage of this mitochondrial contig reach 499.92-fold. Blastn searches against the IDBA-tran assembly identify 19 possible mitochondrial contigs. Twelve contigs with a length larger than 1,000 bp match the 137,775 bp mitochondrial contig assembled by MITObim. Of which, five largest contigs have the length of 42,635, 20,394, 16,519, 7,563, and 5,602 bp, respectively. The IDBA-tran assembly confirms the result from MITObim to some extent. Considering the completeness of the mitochondrial genome assembled, the following sections will focus on the result from the assembler MITObim.

### General Features of *B. sorokiniana* Mitochondrial Genome

The *B. sorokiniana* mtDNA sequence shows similar values concerning AT content, AT skew, and GC skew to most other dothideomycete fungi (mean AT content of 70.5%, mean AT skew of 0.0043, and mean GC skew of 0.0203). The mean AT content of the complete mitochondrial genome calculated for *B. sorokiniana* is 69.5%. The AT skew is slightly negative (-0.0043), while the GC skew is positive (0.0263). These results suggest that there is no significant strand-specific bias of nucleotide composition in the mitochondrial genome of *B. sorokiniana*. The gene content of *B. sorokiniana* mitochondrial genome is characteristic of fungal mitochondria (Figure 1 and Supplementary Table S2), including the usual set of genes interrupted by numerous introns (up to 60,277 bp in length) and separated by long intergenic spacers (up to 53,712 bp in length). A total of 12 core mitochondrial protein-coding genes are identified. In addition to the core protein-coding genes, 52 free-standing open reading frames of unknown function (uORFs) are annotated. The remaining conserved genes identified include two ORFs coding for the putative ribosomal protein S3 (*rps3*) gene and the putative ribosomal protein S5 (*rps5*) gene, the fungal mitochondrial RNase P (*rnpB*) gene, 38 tRNA genes and two mitochondrial ribosomal RNA genes (*rrnL* and *rrnS*).

### Protein-Coding Genes and Codon Usage

The 12 core protein-coding genes (*atp6*, *cob*, *cox1*, *cox2*, *cox3*, *nad1*, *nad2*, *nad3*, *nad4*, *nad4L*, *nad5*, and *nad6*) are typical to fungal mtDNA, which are related to the mitochondrial oxidative phosphorylation pathway. The *atp8* gene is missing, which resembles that in the mitochondrial genome of *B. cookei*. Five protein-coding genes (*cox1*-*3* and *nad2*-*3*) are coded at the light strand, while the remaining seven ones are coded at the heavy strand (Figure 1). Eight out of 12 protein-coding genes are interrupted by introns, namely *atp6*, *cob*, *cox1*, *cox3*, *nad1*, *nad2*, *nad4*, and *nad5*. The highest number of intron sequences (8) are present in the *cox1* gene.

In the core protein-coding genes of *B. sorokiniana*, Ile (I), Leu2 (L2), and Phe (F) are among the most frequently found amino acids with the frequency of AUU (14.8%), AUA (5.48%), and AUC (1.40%) for Ile, UUA (10.17%) and UUG (0.82%) for Leu2, UUU (5.63%) and UUC (2.65%) for Phe, respectively. In *B. sorokiniana* mitochondrial genome, 82.33% codons terminate with A or T. The codon usage pattern can be due to the relatively high AT content found in the whole mitochondrial genome. The average AT content of the core protein-coding genes is 70.5%, and the third codon positions have the highest AT content (82.3%).

### Ribosomal Protein Genes and Mitochondrial RNase P Gene

The *rps3* gene and the *rps5* gene are found between *cox3* and *nad1*, with the lengths of 399 and 555 bp, respectively. Both genes have the same positions and the similar sequence lengths as those in the mitochondrial genome of *B. cookei*. The genes of *rps3* and *rps5* are coded at the light strand. The AT content of *rps3* gene and *rps5* gene are 78.9 and 73.6%, which are higher than that of the core protein-coding genes. In addition, the fungal mitochondrial RNase P gene (*rnpB*) is identified between *trnP*_2 and *trnV*_2, with a length of 563 bp. The AT content of *rnpB* gene is 73.4%.

### Transfer RNA and Ribosomal RNA Genes

A total of 38 tRNA genes are identified in the mitochondrial genome of *B. sorokiniana* coding for 22 amino acids (Supplementary Figure S1), with length ranging from 68 bp (*trnC*_1) to 85 bp (*trnY*). Seven tRNA genes (*trnC*, *trnH*, *trnL1*, *trnL2*, *trnP*, *trnQ*, and *trnV*) are duplicated. Four *trnM* genes are present in the mitochondrial genome, as the genes of *trnN* and *trnR*. The majority of tRNA genes are located between *rrnS* and *nad4*, and coded at the heavy strand in an anti-clockwise direction. No introns have been found in tRNA genes. All tRNA genes can be folded into the cloverleaf secondary structure. However, the *trnC*_1 displays an unusual acceptor stem due to nucleotide missing. In addition, the anticodon loop in *trnP*_1 is replaced with a large loop structure due to redundant nucleotides.

Two mitochondrial rRNA genes are identified, namely *rrnL* gene and *rrnS* gene. The *rrnL* gene is 4,440 bp in length, which is placed between *trnP_*1 and *trnT*. The *rrnL* gene is separated into two fragments by an intronic spacer with length of 273 bp. The *rrnS* gene is 1,659 bp, and positioned between *trnR_*2 *and trnL2_*2. The *rrnS* is a complete gene sequence, without intronic spacer identified in this gene. The AT content of *rrnL* and *rrnS* gene are 65.1 and 63.3%, respectively.

### Phylogenetic Inference

Partition homogeneity tests showed the dataset of PCG_nt to be congruent with the dataset of rrn (*P* = 0.13). Therefore, the alignments of PCG_nt and rrn can be combined. The trn alignment is significantly incongruent when evaluated against PCG_nt (*P* = 0.01) or rrn (*P* = 0.01).

The separate ML analyses for individual protein-coding genes (Supplementary Table S3) recover three order groups, namely, *Capnodiales*, *Botryosphaeriales* and *Pleosporales*. The *Venturiales* is often a deeply-diverging clade, while the *Pleosporales* is placed in a relatively derived position. Despite with these results, the relationships among orders vary greatly across separate analyses. The discordance indicates the conflicting signals in the individual protein-coding gene data.

**FIGURE 2:**
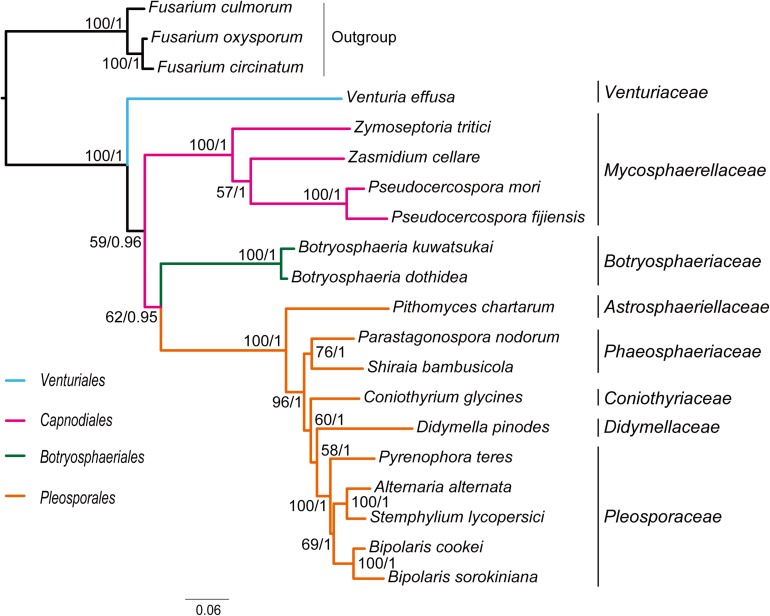
Maximum likelihood tree inferred from the dataset of PCG-rrn using IQ-TREE, under the partition schemes and best-fitting models selected by PartitionFinder 2. Node numbers show bootstrap support values (left) and poster probability values from MrBayes (right).

**FIGURE 3:**
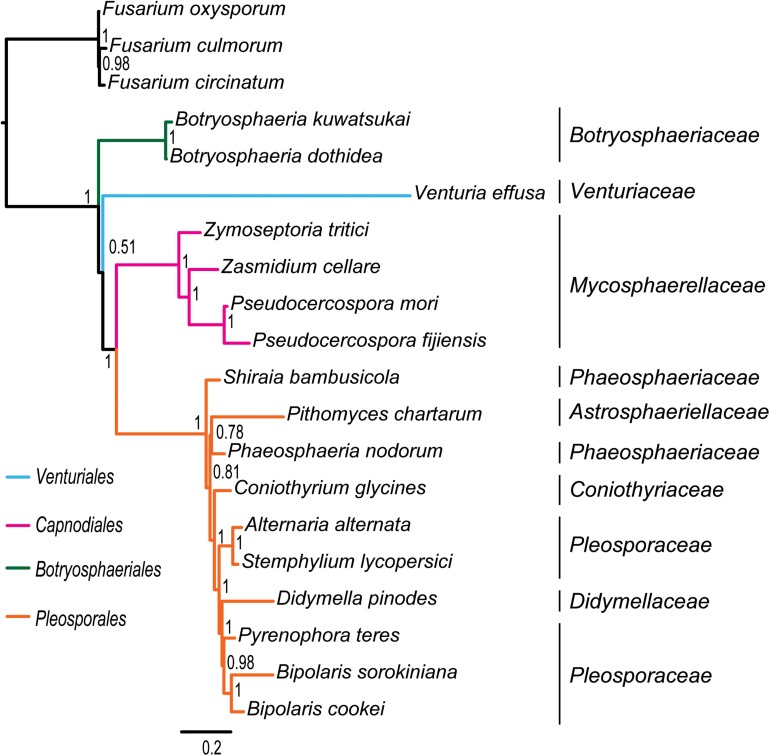
Bayesian tree inferred from the dataset of PCG_aa using PhyloBayes, under the site-heterogeneous CAT-MTZOA model. Node numbers show the poster probability values.

**FIGURE 4:**
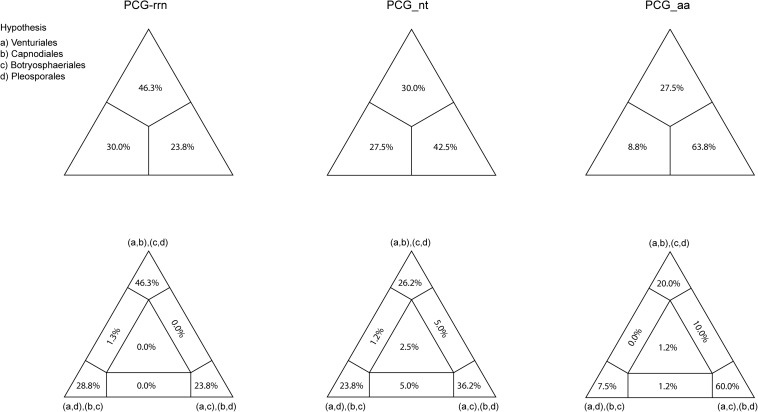
Results obtained from the four-cluster likelihood-mapping analyses on the datasets of PCG-rrn, PCG_nt, and PCG_aa. The triangles above are the three posterior probabilities for the three possible hypotheses; and the triangles down are the two-dimensional simplex graphs supporting different evolutionary information.

Combined protein-coding gene datasets (i.e., PCG_nt and PCG_aa) and the PCG-rrn dataset also recovered the three order-level lineages outlined above (Figures 2, 3 and Supplementary Figures S2–S4). Compared to separate ML analyses on individual protein-coding genes, combined analyses yield higher support values for main nodes in Dothideomycetes. Based on the PCG-rrn data, ML analysis and MrBayes analysis result in an identical tree topology (Figure 2). In which, the *Venturiales* is supported as the sister group to all other Dothideomycetes. The *Botryosphaeriales* is placed as sister group to *Pleosporales* (*BP* = 62, *PP* = 0.95). This arrangement receive 46.3% quartet support from the FcLM analysis on PCG-rrn data (Figure 4). The datasets of PCG_nt and PCG_aa show weaker signal for this relationship (30% of quartets from PCG_nt, and 27.5% of quartets from PCG_aa). Within *Pleosporales*, the family *Astrosphaeriellaceae* is the first clade to diverge, followed by the families *Phaeosphaeriaceae* and *Coniothyriaceae* in a sequential order. The *Pleosporaceae* forms a sister group to *Didymellaceae*. Within *Pleosporaceae*, our analyses maximally support *B. sorokiniana* as a sister group to *B. cookei.* This sister group relationship is also recovered in the analyses of PCG_nt and PCG_aa (*BP* = 100, *PP* = 1), irrespective of method of phylogenetic inference.

Under the site-homogeneous models, trees obtained from the analyses of PCG_nt data (Supplementary Figures S2A,B) are identical in the major lineages to those from the PCG-rrn analyses, but varied in the relationships among orders and families. The similar patterns are revealed in the analyses of PCG_aa data (Supplementary Figures S3A,B). The ML tree and MrBayes tree inferred from the amino acid data of PCG_aa differ from the nucleotide data of PCG-rrn in that the *Capnodiales* is retrieved as the sister group to *Pleosporales*. This sister group relationship is also recovered in the ML analysis from the nucleotide data of PCG_nt (Supplementary Figure S2A). The FcLM results show weak to moderate signal for the placement of *Capnodiales* as sister group to *Pleosporales* (23.8, 42.5, and 63.8% of quartets, PCG-rrn, PCG_nt, and PCG_aa, respectively).

Under the site-heterogeneous models, phylogenetic trees resulting from PhyloBayes analyses based on datasets of PCG_aa, PCG_nt and PCG-rrn consistently place *Botryosphaeriales* as the first diverging clade in Dothideomycetes (Figure 3 and Supplementary Figures S4A,B). In the PhyloBayes trees of PCG_nt and PCG-rrn, *Venturiales* and *Capnodiales* are recovered as sister group. But this relationship lacks significant support from both datasets (*PP* ≤ 0.62). By contrast, *Venturiales* is sister to a clade comprising *Capnodiales* and *Pleosporales* in the PhyloBayes trees from PCG_aa (Figure 3). Moreover, the *Capnodiales* is supported as a sister-group of *Pleosporales* (*PP* = 1). Within *Pleosporales*, the *Phaeosphaeriaceae* is non-monophyletic. Both *B. sorokiniana* and *B. cookei* are consistently grouped together with strong support (*PP* = 1).

**FIGURE 5:**
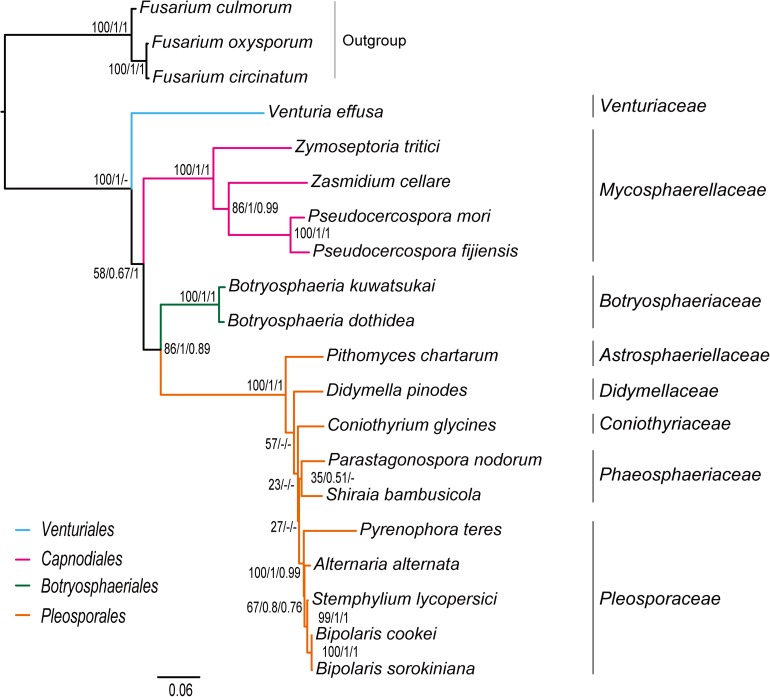
Maximum likelihood tree inferred from the dataset of rrn using IQ-TREE. Node numbers show bootstrap support values (left), poster probability values from MrBayes (middle), and poster probability values from PhyloBayes (right).

**FIGURE 6:**
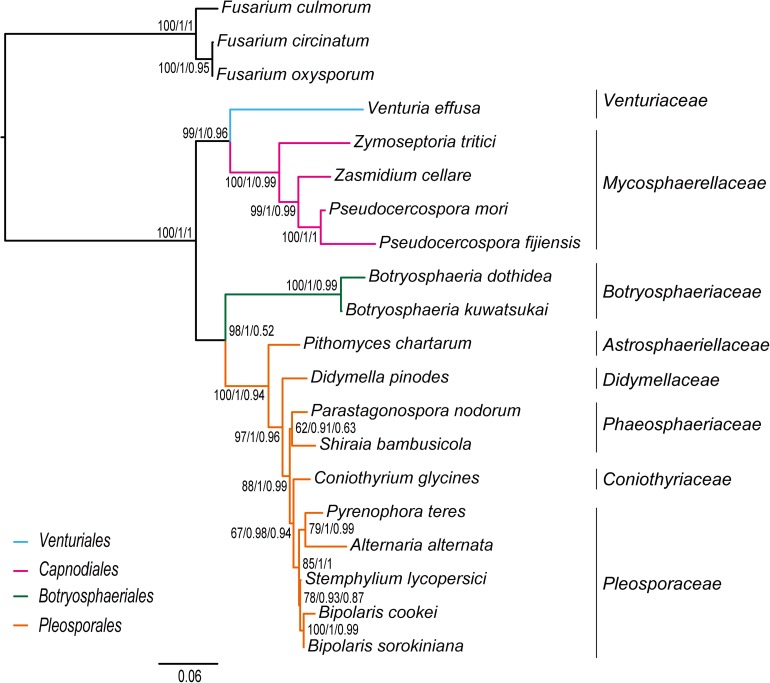
Maximum likelihood tree inferred from the dataset of trn using IQ-TREE. Node numbers show bootstrap support values (left), poster probability values from MrBayes (middle), and poster probability values from PhyloBayes (right).

The inference methods do not change the tree topology greatly in the phylogenetic analyses based on the datasets of rrn and trn (Figures 5, 6). Under the ML and MrBayes inferences, the rrn data recovers a very similar inter-order relationship as the PCG-rrn data. The *Venturiales* is the deepest lineage in Dothideomycete, while *Botryosphaeriales* forms a sister-group of *Pleosporales* (*BP* = 86 and *PP* = 1 in Figure 5). The trn data also supports a sister-group relationship between *Botryosphaeriales* and *Pleosporales* (*BP* = 98 and *PP* = 1 in Figure 6). But it recovers *Venturiales* and *Capnodiales* as sister group, which is not favored in analyses from rrn data. The PhyloBayes trees from the rrn and trn datasets show very similar topological structures as those in the ML and MrBayes trees, but with lower support values for several nodes. The results of the FcLM analyses on the rrn and trn datasets strongly support the (*Botryosphaeriales*, *Pleosporales*) clade (Supplementary Figure S5).

## Discussion

Thus far, the complete mtDNA sequences available in Dothideomycete are scarce. Compared to animal mitochondrial genomes, fungal mitochondrial genomes have some characteristic features. In particular, presence of numerous introns within the fungal mitochondrial protein-coding genes makes it difficult to align sequences. This study presents an effective procedure to deal with this problem. Most of the nodes in our phylogenies have maximal statistical support, demonstrating the utility of our approach for generating mitochondrial genome data matrix useful in resolving the relationships in Dothideomycetes.

In addition to the enormous variations in size of mitochondrial genomes, the gene content and gene order may be different among fungal species. The gene content and gene order of the *B. sorokiniana* mitochondrial genome are very similar to those of *B. cookei* (Zaccaron and Bluhm, 2017). The major difference is the occurrence of a variable number of tRNA genes. There are only 28 tRNA genes present in the mitochondrial genome of *B. cookei*, in contrast with 38 tRNA genes in *B. sorokiniana.* Besides tRNA genes, the numbers of unique ORFs, introns inserted in the protein-coding genes and intergenic spacers are different between two *Bipolaris* species. Compared with animal mitochondrial genomes, the *atp9* is an additional gene occurring in many fungi (Paquin et al., 1997; Losada et al., 2014; Salavirta et al., 2014; Torriani et al., 2014; Lin et al., 2015; Ellenberger et al., 2016; Kanzi et al., 2016; Kang et al., 2017; Deng et al., 2018). However, the *atp9* gene is missing in *B. sorokiniana* and in *B. cookei.* Although plasmids and plasmid-like elements have been reported from mitochondria and mitochondrial genomes in some fungal species (Griffiths, 1995; Sakurai et al., 2004; Formighieri et al., 2008; Monteiro-Vitorello et al., 2009), our sequence analysis detected no apparent linear or circular plasmids in *B. sorokiniana*.

Mitochondrial phylogenomic analyses have been extensively used in the studies of animals (Boore, 1999; Garesse and Vallejo, 2001; Xu et al., 2006; Mulcahy et al., 2012; Cameron, 2014; Li et al., 2017; Du et al., 2019; Song F. et al., 2019; Song N. et al., 2019; Tang et al., 2019; Klucnika and Ma, 2020), but few attempts to produce fungal phylogenies based on mitochondrial genome sequences. Dothideomycetes is the largest class of Ascomycota (Hyde et al., 2013), which includes many economically significant plant pathogens. Phylogeny of the Dothideomycetes has attracted systematists’ attention in recent years (Schoch et al., 2006, 2009a,b; Shearer et al., 2009; Suetrong et al., 2009; Nelsen et al., 2011a, b; Zhang et al., 2011; Liu et al., 2017). Some authors have initiated studies using single-gene and multi-gene phylogenetic analyses to investigate the relationships of Dothideomycetes (Schoch et al., 2006, 2009a; Shearer et al., 2009; Suetrong et al., 2009; Hyde et al., 2013). These molecular studies more frequently applied nuclear gene sequence data (e.g., nuc SSU rDNA, nuc LSU rDNA, EF1-α and ITS) to establish the phylogenetic framework (Schoch et al., 2006, 2009a,b; Shearer et al., 2009; Hyde et al., 2013; Manamgoda et al., 2012, 2014). Nuclear genes have different mechanisms of inheritance in comparison with mitochondrial genes. In general, nuclear genes show slower rates of evolution than mitochondrial genes (Brown et al., 1979; Moriyama and Powell, 1997; Johnson and Clayton, 2000; San Mauro et al., 2004). Comparisons of phylogenies based on nuclear and mitochondrial gene sequences are essential to our understanding of the phylogenetic affinities of Dothideomycetes.

Traditionally, members of *Venturiaceae* were classified in the order *Pleosporales* (Hyde et al., 2013). Schoch et al. (2009a, b) recovered *Venturiaceae* as a separated clade from the core members of *Pleosporales*, with nuclear gene fragments data. Based on combined analysis of molecular, morphological and ecological evidence, Zhang et al. (2011) established the order *Venturiales* comprising *Venturiaceae* and *Sympoventuriaceae*. Our phylogenetic estimates on the mitochondrial genome sequence data consistently support the *Venturiaceae* as an independent clade. The *Botryosphaeriales* is another recently proposed order (Schoch et al., 2006). Based on the PCG-rrn data, ML and MrBayes analyses returned a sister-group relationship between *Botryosphaeriales* and *Pleosporales*. The earlier molecular studies based on the nuclear gene sequences also suggested a relatively close affiliation of *Botryosphaeriales* to *Pleosporales* (Schoch et al., 2006; Hyde et al., 2013). Concerning the deep relationships among four orders surveyed, the branching pattern of {*Venturiales*, [*Capnodiales*, (*Botryosphaeriales*, *Pleosporales*)]} inferred from the PCG-rrn data and the rrn data is congruent with the multi-locus phylogenies from nuclear genes (Schoch et al., 2009a; Hyde et al., 2013).

**FIGURE 7:**
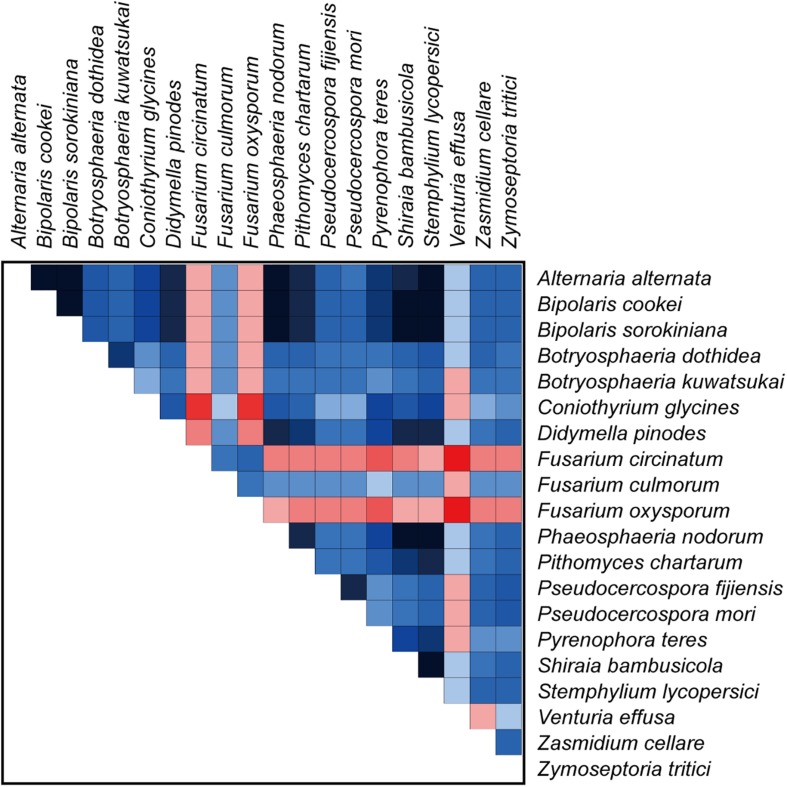
The AliGROOVE graph shows the mean similarity scores between sequences, based on the analysis of the *rrnL* gene alignment. AliGROOVE scores range from −1 (indicating great difference in rates from the remainder of the data set, i.e., red coloring implies the significant heterogeneity) to +1 (indicating rates match all other comparisons). The results from AliGROOVE analyses based on other datasets are shown in Supplementary Figure S6.

Our analyses using a variety of datasets and different inference methods largely recover the same substantial clades in Dothideomycetes, albeit with discordant relationships between them. No significant saturation was found in any data type alignments (Iss < Iss.cSym and Iss < Iss.cAsym in Supplementary Table S4). The presence of heterogeneous sequences and lineage-specific substitution rates may provide some explanation for the incongruence between gene trees. The instability of the phylogenetic placement of *Venturiales* among analyses deserves special attention. This clade displays the obviously long branch length when compared with other dothideomycete lineages. The sequence heterogeneity analyses revealed that *V. effusa* has the notably higher heterogeneity than other dothideomycete species in the matrix of *rrnL* gene sequences (Figure 7). The analyses of synonymous substitution and non-synonymous substitution showed that the *Venturiales* has the elevated frequencies of synonymous substitutions and non-synonymous substitutions (Table 2). Both factors may lead to random similarity associated with long-branch effect. In addition to *V. effusa*, four species from *Capnodiales* (*Zymoseptoria tritici*, *Zasmidium cellare*, *Pseudocercospora mori*, and *Pseudocercospora fijiensis*) also have higher synonymous and non-synonymous substitution rates (Table 2). Therefore, the grouping of *Venturiales* and *Capnodiales* might be the result of long-branch attraction.

**TABLE 2:** The synonymous and non-synonymous nucleotide substitutions calculated for each species.

Species	*Ks*	*Ka*
*Alternaria alternata*	0.9016	0.1116
*Bipolaris cookei*	0.8452	0.1062
*Bipolaris sorokiniana*	0.8601	0.1312
*Botryosphaeria dothidea*	1.1755	0.1492
*Botryosphaeria kuwatsukai*	1.1546	0.1516
*Coniothyrium glycines*	0.8940	0.1091
*Didymella pinodes*	1.0238	0.1372
*Fusarium circinatum*	1.0087	0.1928
*Fusarium culmorum*	0.9808	0.1981
*Fusarium oxysporum*	0.9953	0.1923
*Phaeosphaeria nodorum*	0.9286	0.1066
*Pithomyces chartarum*	0.9670	0.1301
*Pseudocercospora fijiensis*	1.2034	0.1578
*Pseudocercospora mori*	1.2606	0.1452
*Pyrenophora teres*	0.9070	0.1114
*Shiraia bambusicola*	0.9158	0.1109
*Stemphylium lycopersici*	0.8808	0.1105
*Venturia effusa*	1.4006	0.1979
*Zasmidium cellare*	1.1401	0.1500
*Zymoseptoria tritici*	1.5408	0.1484

**Ks*, number of synonymous substitutions per synonymous site; *Ka*, number of non-synonymous substitutions per non-synonymous site.*

Due to rapid evolution of mtDNA (Brown et al., 1979; Simon et al., 2006), long branches occur frequently in the phylogenetic analyses based on mitochondrial genome sequences (Li et al., 2015; Song et al., 2016, Song N. et al., 2019; Timmermans et al., 2016; Liu et al., 2018; Tang et al., 2019). Long branches may have a negative effect on the accuracy of estimation of phylogenetic relationships. Previous studies (Lartillot et al., 2007; Li et al., 2015; Liu et al., 2018; Song F. et al., 2019; Song N. et al., 2019) showed that the site-heterogeneous model implemented in PhyloBayes could reduce the effects of compositional and mutational bias, and further suppress the long-branch attraction artifacts in the animal phylogeny. However, analyzing the current fungal mitochondrial genome data under different models did not resolve the observed incongruence. The PhyloBayes analysis on amino acid data under site-heterogeneous model breaks the grouping of *Venturiales* and *Capnodiales*, and recovers the *Pleosporales* as a sister group to *Capnodiales* (Figure 3). The PhyloBayes analyses on nucleotide datasets (PCG_nt and PCG-rrn) still retrieve a sister group of *Venturiales* and *Capnodiales* (Supplementary Figures S4A,B).

In contrast to varied results from the datasets compiled by protein-coding genes, consistency between phylogenetic reconstructions at deep level relationships within Dothideomycetes was remarkably improved by the datasets of rRNA gene and tRNA gene. The variable rates of molecular evolution in different gene sequences introduce a significant source of conflict. Slow evolving genes are more suitable for resolving deep level phylogenies (Donoghue and Sanderson, 1992; Giribet, 2002; Nosenko et al., 2013). Rates of evolutionary substitution in rRNA or tRNA genes are unusually lower than those for protein-coding genes (Ochman and Wilson, 1987; Yamamoto and Harayama, 1998; Nosenko et al., 2013). Thus, rRNA or tRNA data partitions are likely to give reliable estimates of the phylogenetic relationships among dothideomycete orders.

The lack of resolution in phylogenetics may be attributed to low levels of phylogenetic signal or highly conflicting phylogenetic signal (Whitfield and Kjer, 2008; Suh, 2016). Our FcLM analyses showed substantial levels of phylogenetic conflict for the interrelationships of four dothideomycete orders. In addition, deficient taxon sampling can also lead to poor resolution of phylogenetic relationships (Nabhan and Sarkar, 2012). We acknowledge the sensitivity of taxon sampling to phylogenetic reconstructions of a highly diverse fungal lineage. Yet, the effectively analytical approaches presented in this study are expected to inspire more mitochondrial phylogenomic analyses of Dothideomycetes. Increasing the available mitogenomic data will inevitably contribute to addressing the persisting phylogenetic uncertainties in this important fungal group.

## Conclusion

We sequenced and annotated the complete mitochondrial genome of *B. sorokiniana*, which is only the second mitochondrial genome sequence published in the genus *Bipolaris*. The gene content and organization are similar to those of the *B. cookei*. The large number and size of introns and intergenic spacers result in the large genome size. Several order- and family-level taxa are robustly supported by the current mtDNA sequence data. The *Venturiaceae* is consistently recovered to be an independent clade and phylogenetically distant from *Pleosporales*. This result confirms the view of prior studies (Schoch et al., 2009a, b; Zhang et al., 2011). Incongruence between phylogenies constructed using various data types may stem from conflicting signals and unequal evolutionary rates. Tremendous advances of new DNA sequencing technologies have opened a window into the field of phylogenomic research, which will provide more alternative methods to tackle the problems. An understanding of mitochondrial genome evolution and phylogenetic relationships within Dothideomycetes would require the sequencing of mitochondrial genomes from more members of this group.

## Data Availability Statement

The datasets generated for this study can be found in the GenBank under the accession number MN978926.

## Author Contributions

NS and YG conceived this study. NS analyzed the data, prepared the figures, and drafted the manuscript. YG and XL participated in early analysis of preliminary data and manuscript writing and revision. YG provided suggestion for the research, contributed to the data interpretation, writing, and revising the manuscript critically. All authors have read and approved the final version of the manuscript.

## Conflict of Interest

The authors declare that the research was conducted in the absence of any commercial or financial relationships that could be construed as a potential conflict of interest.
