# Supplementary material for: The Mitochondrial Genome of the Phytopathogenic Fungus Bipolaris sorokiniana and the Utility of Mitochondrial Genome to Infer Phylogeny of Dothideomycetes
Source: Front Microbiol. 2020 May 8;11:863. doi: 10.3389/fmicb.2020.00863 (PMC7225605; doi:10.3389/fmicb.2020.00863)
Supplement: Supplementary file 6 [file Data_Sheet_2.pdf]

Supplementary Material

Figure legends

Figure S1. Putative secondary structures of the 38 tRNA genes from *Bipolaris sorokiniana*.

Watson-Crick pairs is indicated by lines, and wobble G-U pairs is indicated by dots. The non-canonical pairs are not marked.

Figure S2. Phylogenetic trees inferred from the dataset of PCG_nt, under ML and MrBayes analyses.

(A) ML tree inferred from PCG_nt, under the partition schemes and best-fitting models selected by PartitionFinder 2. Node numbers show bootstrap support values. **(B)** MrBayes tree inferred from PCG_nt, under the partition schemes and best-fitting models selected by PartitionFinder 2. Node numbers show poster probability values.

Figure S3. Phylogenetic trees inferred from the dataset of PCG_aa, under ML and MrBayes analyses.

(A) ML tree inferred from PCG_aa, under the partition schemes and best-fitting models selected by PartitionFinder 2. Node numbers show bootstrap support values. **(B)** MrBayes tree inferred from PCG_aa, under the partition schemes and best-fitting models selected by PartitionFinder 2. Node numbers show poster probability values.

Figure S4. PhyloBayes trees inferred from the datasets of PCG_nt and PCG-rrn.

(A) PhyloBayes trees from PCG_nt, under the site-heterogeneous CAT-GTR model. Node numbers show poster probability values. **(B)** PhyloBayes trees from PCG-rrn, under the site-heterogeneous CAT-GTR model. Node numbers show poster probability values.

Figure S5. Results obtained from the four-cluster likelihood-mapping analyses on the datasets of rrn and trn.

The triangles above are the three posterior probabilities for the three possible hypotheses; and the triangles down are the two-dimensional simplex graphs supporting different evolutionary information.

Figure S6. The AliGROOVE analyses were performed based on the datasets of PCG-rrn, PCG_nt, PCG_aa, *rrnS* and trn, respectively.

AliGROOVE scores range from -1 (indicating great difference in rates from the remainder of the data set, i.e. red coloring implies the significant heterogeneity) to +1 (indicating rates match all other comparisons).
