# Supplementary material for: The Mitochondrial Genome of the Phytopathogenic Fungus Bipolaris sorokiniana and the Utility of Mitochondrial Genome to Infer Phylogeny of Dothideomycetes
Source: Front Microbiol. 2020 May 8;11:863. doi: 10.3389/fmicb.2020.00863 (PMC7225605; doi:10.3389/fmicb.2020.00863)
Supplement: Supplementary file 7 [file Presentation_1.PDF]

The commands used in the selectSeqs.pl script.

```
perl selectSeqs.pl -m atp6 matrix_inc-all-genes.fas > atp6_matrix.fas
perl selectSeqs.pl -m cob matrix_inc-all-genes.fas > cob_matrix.fas
perl selectSeqs.pl -m cox1 matrix_inc-all-genes.fas > cox1_matrix.fas
perl selectSeqs.pl -m cox2 matrix_inc-all-genes.fas > cox2_matrix.fas
perl selectSeqs.pl -m cox3 matrix_inc-all-genes.fas > cox3_matrix.fas
perl selectSeqs.pl -m nad1 matrix_inc-all-genes.fas > nad1_matrix.fas
perl selectSeqs.pl -m nad2 matrix_inc-all-genes.fas > nad2_matrix.fas
perl selectSeqs.pl -m nad3 matrix_inc-all-genes.fas > nad3_matrix.fas
perl selectSeqs.pl -m nad4 matrix_inc-all-genes.fas > nad4_matrix.fas
perl selectSeqs.pl -m nad4L matrix_inc-all-genes.fas > nad4L_matrix.fas
perl selectSeqs.pl -m nad5 matrix_inc-all-genes.fas > nad5_matrix.fas
perl selectSeqs.pl -m nad6 matrix_inc-all-genes.fas > nad6_matrix.fas
perl selectSeqs.pl -m rrnL matrix_inc-all-genes.fas > rrnL_matrix.fas
perl selectSeqs.pl -m rrnS matrix_inc-all-genes.fas > rrnS_matrix.fas
perl selectSeqs.pl -m trnA matrix_inc-all-genes.fas > trnA_matrix.fas
perl selectSeqs.pl -m trnC matrix_inc-all-genes.fas > trnC_matrix.fas
perl selectSeqs.pl -m trnD matrix_inc-all-genes.fas > trnD_matrix.fas
perl selectSeqs.pl -m trnE matrix_inc-all-genes.fas > trnE_matrix.fas
perl selectSeqs.pl -m trnF matrix_inc-all-genes.fas > trnF_matrix.fas
perl selectSeqs.pl -m trnG matrix_inc-all-genes.fas > trnG_matrix.fas
perl selectSeqs.pl -m trnH matrix_inc-all-genes.fas > trnH_matrix.fas
perl selectSeqs.pl -m trnI matrix_inc-all-genes.fas > trnI_matrix.fas
perl selectSeqs.pl -m trnK matrix_inc-all-genes.fas > trnK_matrix.fas
perl selectSeqs.pl -m trnL1 matrix_inc-all-genes.fas > trnL1_matrix.fas
perl selectSeqs.pl -m trnL2 matrix_inc-all-genes.fas > trnL2_matrix.fas
perl selectSeqs.pl -m trnM matrix_inc-all-genes.fas > trnM_matrix.fas
perl selectSeqs.pl -m trnN matrix_inc-all-genes.fas > trnN_matrix.fas
perl selectSeqs.pl -m trnP matrix_inc-all-genes.fas > trnP_matrix.fas
perl selectSeqs.pl -m trnQ matrix_inc-all-genes.fas > trnQ_matrix.fas
perl selectSeqs.pl -m trnR matrix_inc-all-genes.fas > trnR_matrix.fas
perl selectSeqs.pl -m trnS1 matrix_inc-all-genes.fas > trnS1_matrix.fas
perl selectSeqs.pl -m trnS2 matrix_inc-all-genes.fas > trnS2_matrix.fas
perl selectSeqs.pl -m trnT matrix_inc-all-genes.fas > trnT_matrix.fas
perl selectSeqs.pl -m trnV matrix_inc-all-genes.fas > trnV_matrix.fas
perl selectSeqs.pl -m trnW matrix_inc-all-genes.fas > trnW_matrix.fas
perl selectSeqs.pl -m trnY matrix_inc-all-genes.fas > trnY_matrix.fas
```

Note: -m “pattern” means extracting the sequences whose name contains the pattern.
