# Supplementary figures and images for: The Mitochondrial Genome of the Phytopathogenic Fungus Bipolaris sorokiniana and the Utility of Mitochondrial Genome to Infer Phylogeny of Dothideomycetes
Source: Front Microbiol. 2020 May 8;11:863. doi: 10.3389/fmicb.2020.00863 (PMC7225605; doi:10.3389/fmicb.2020.00863)

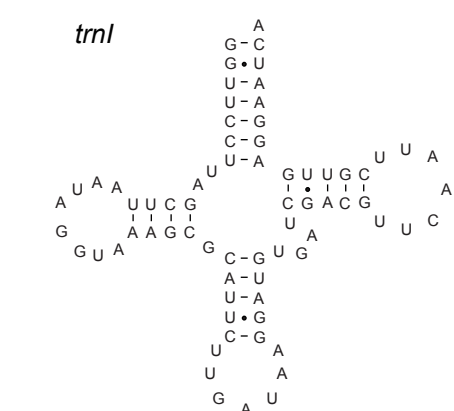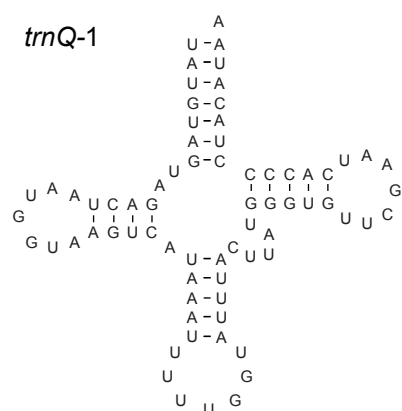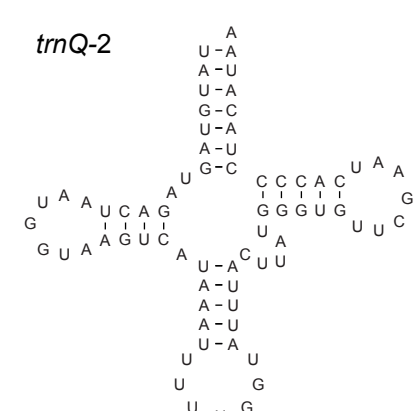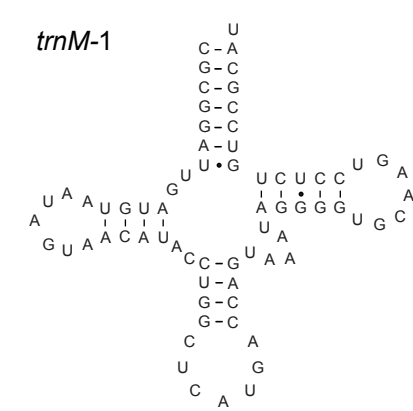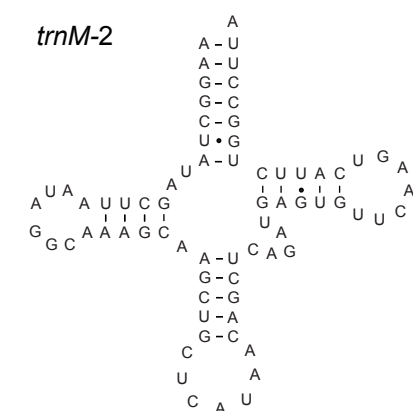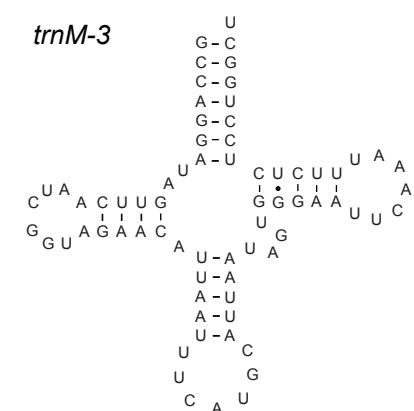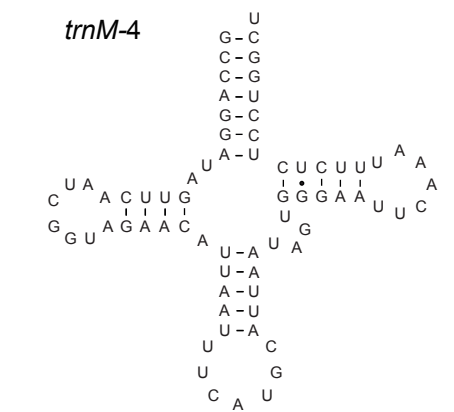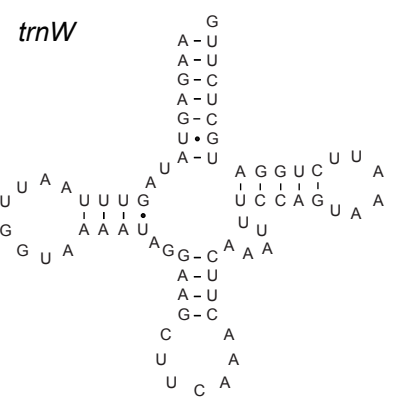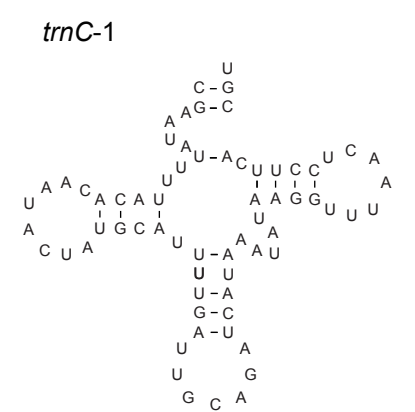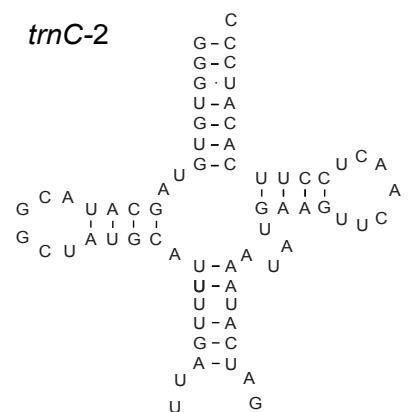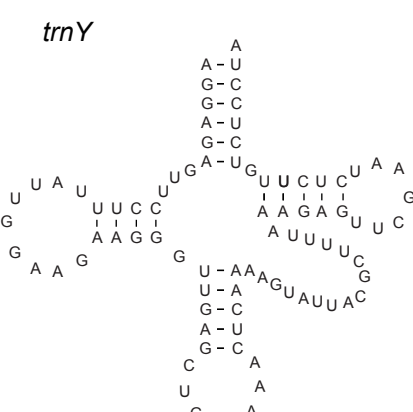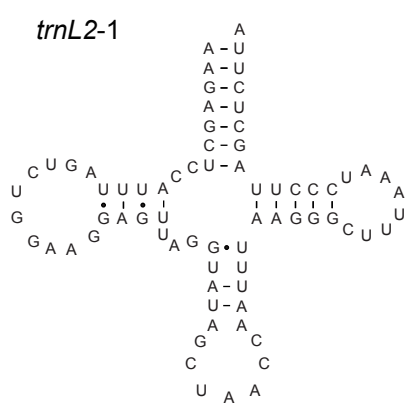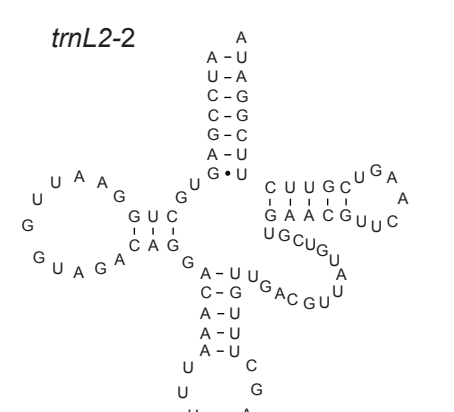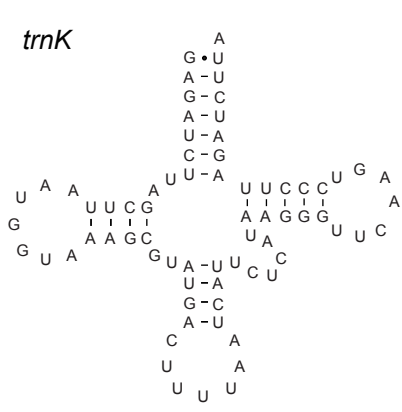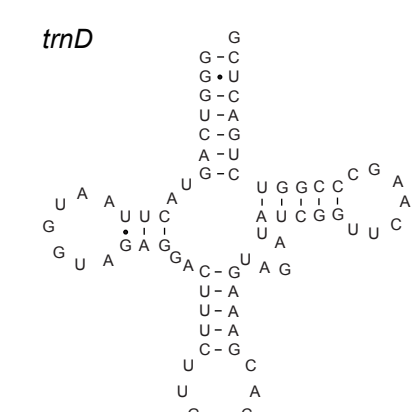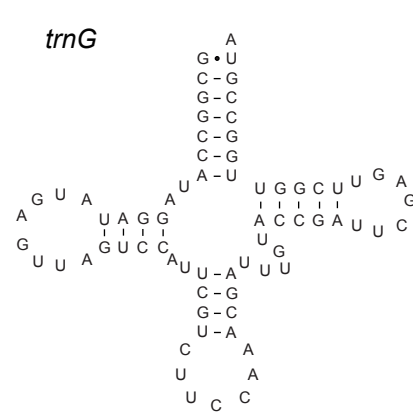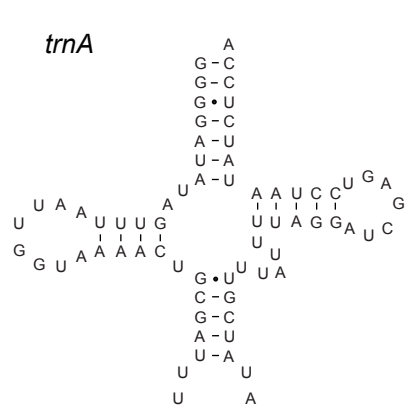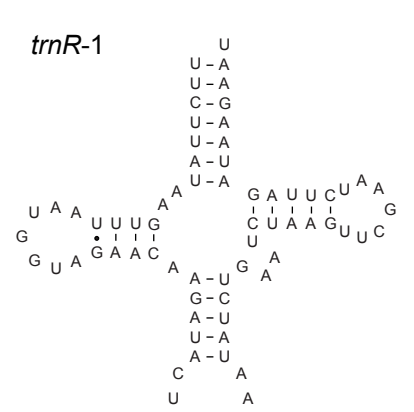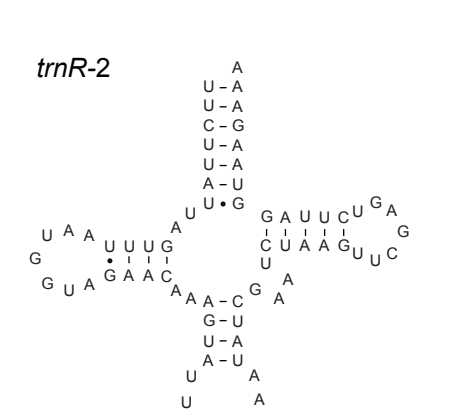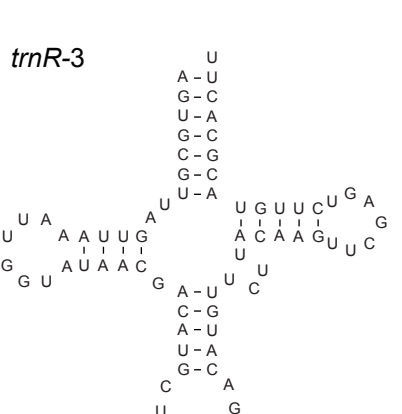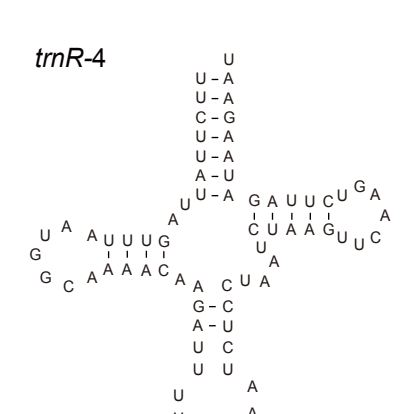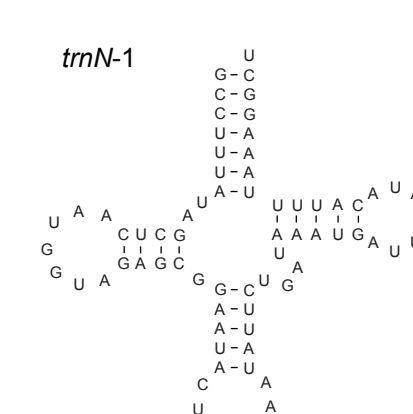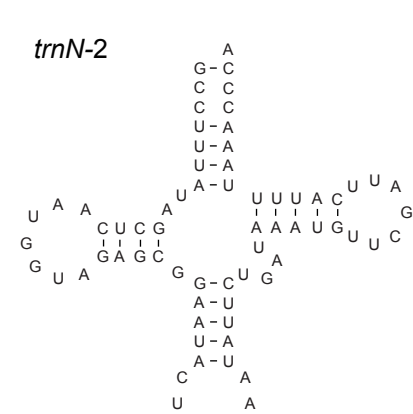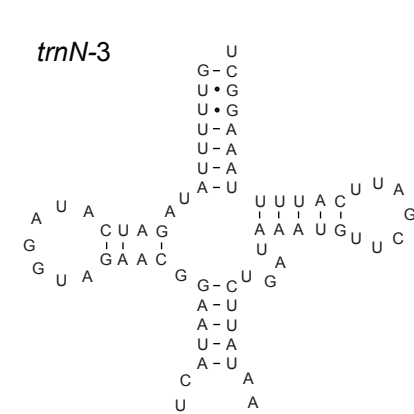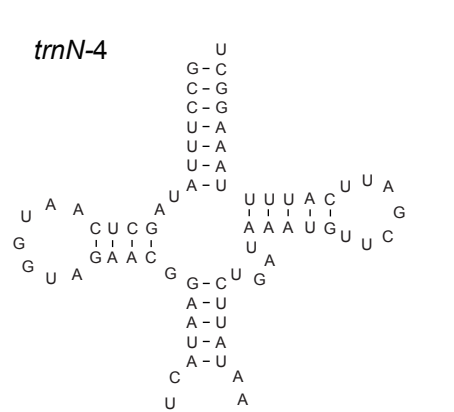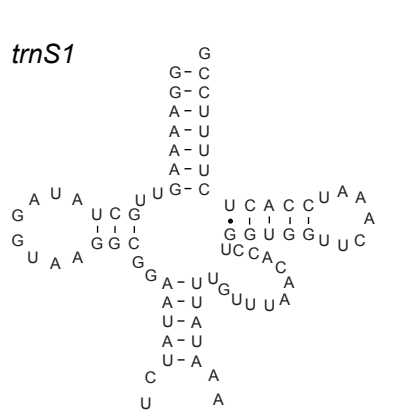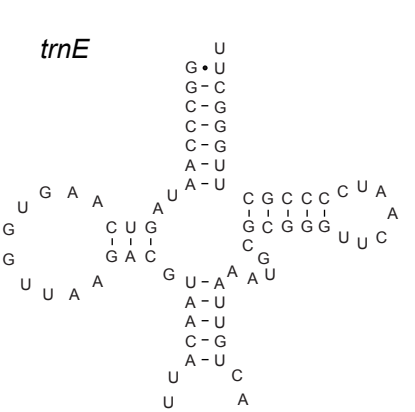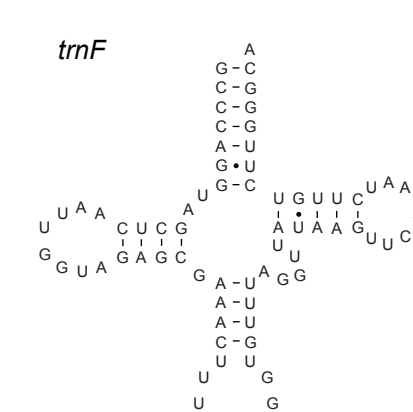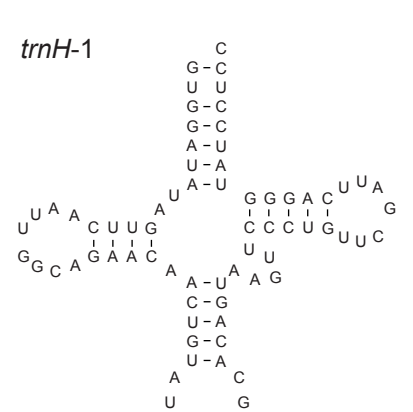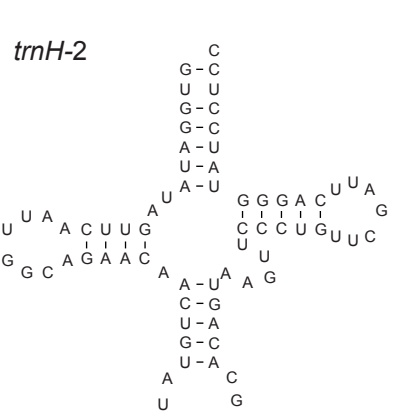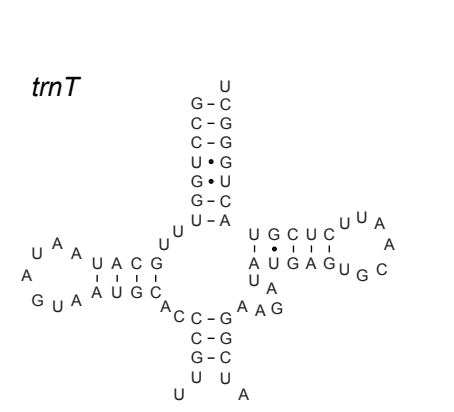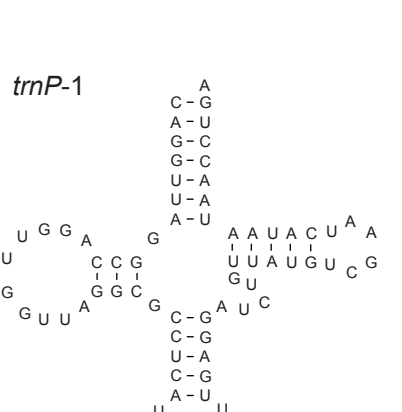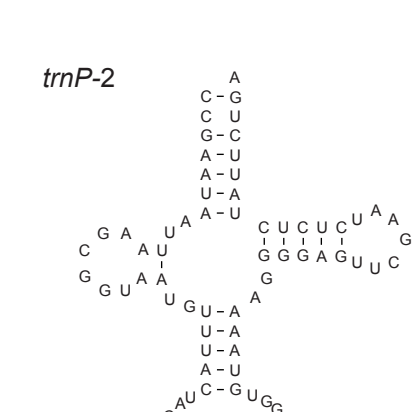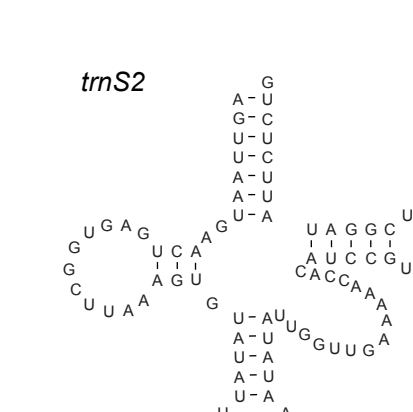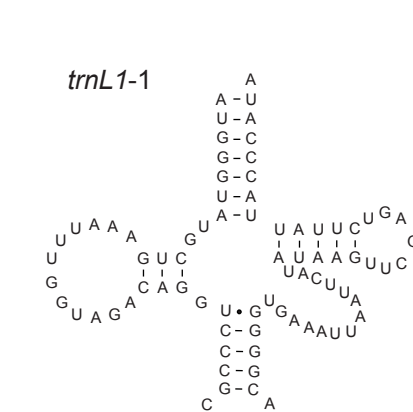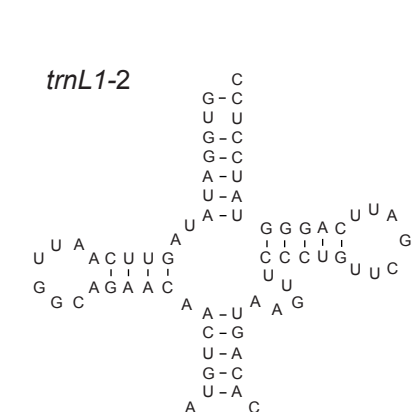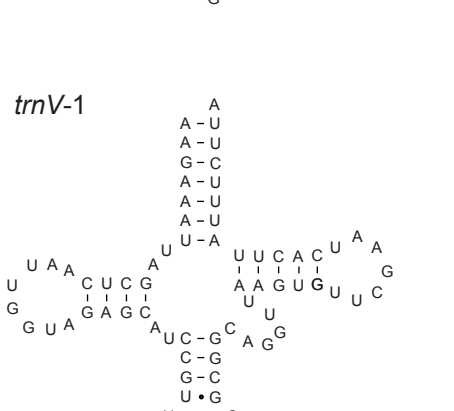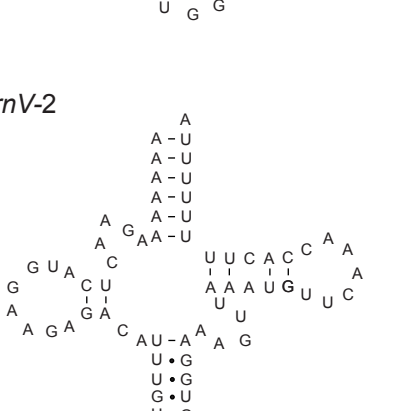

Supplement: Supplementary file 8 [file Image_1.PDF]

(A)

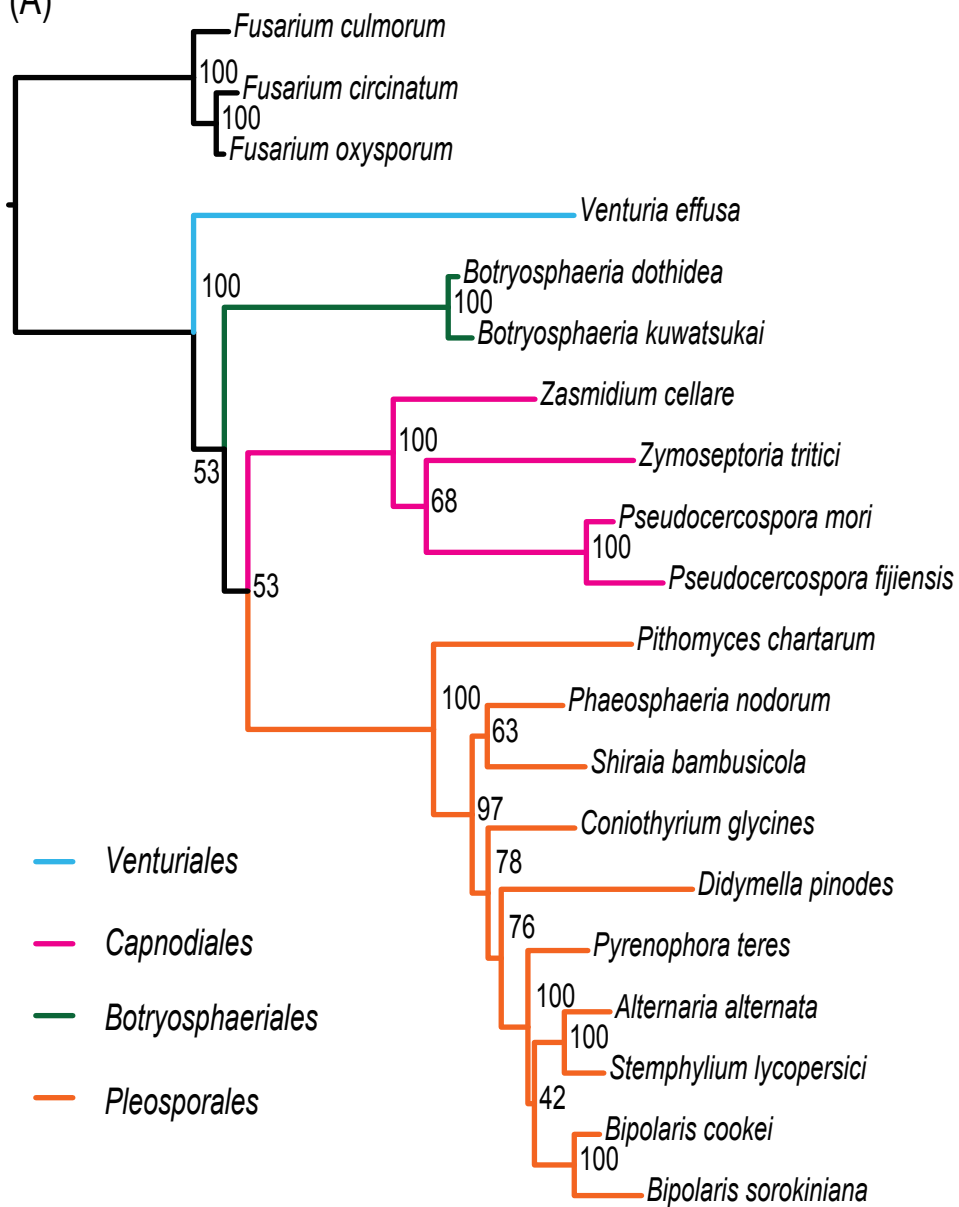

(B)

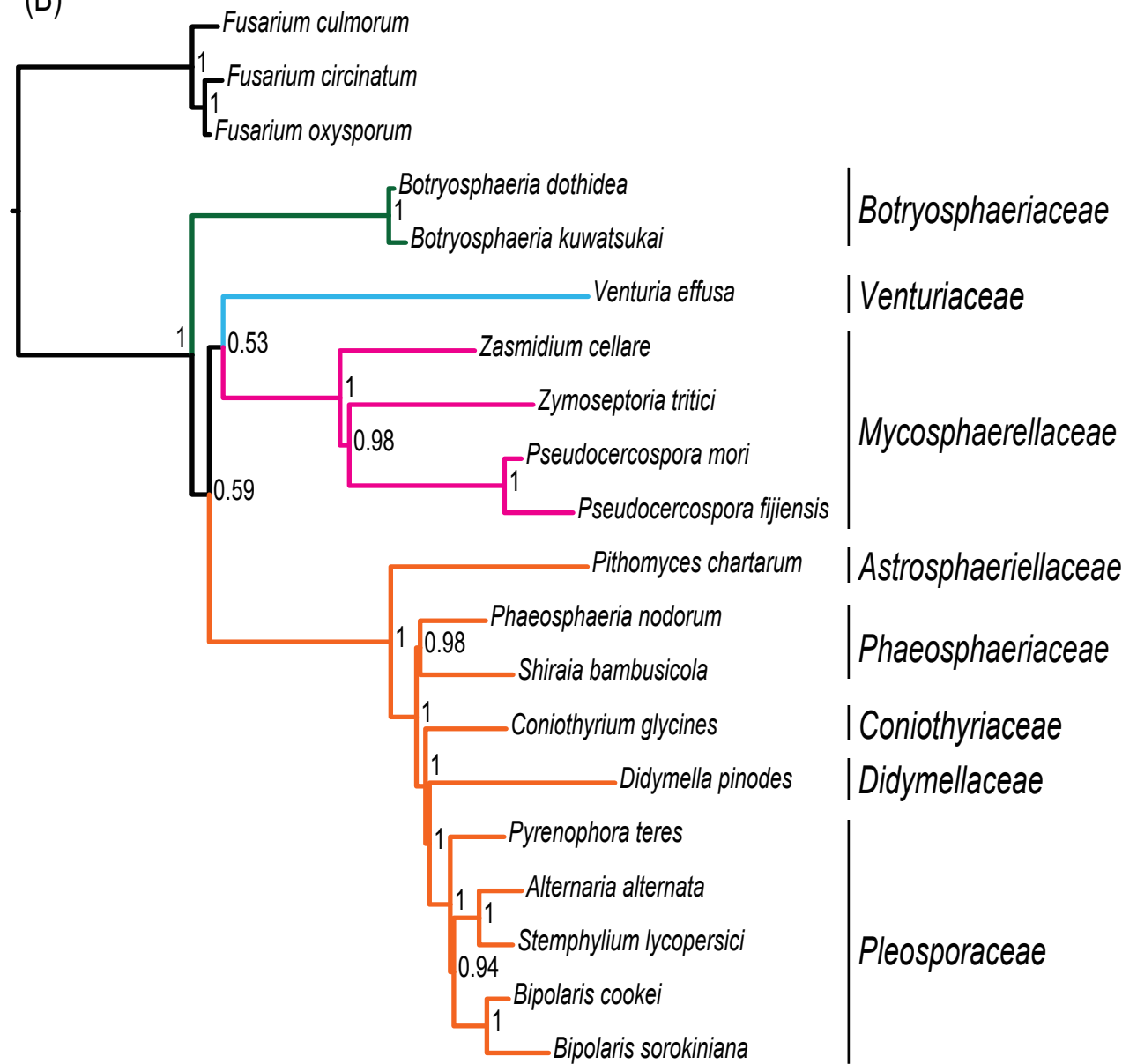

Supplement: Supplementary file 9 [file Image_2.PDF]

(A)

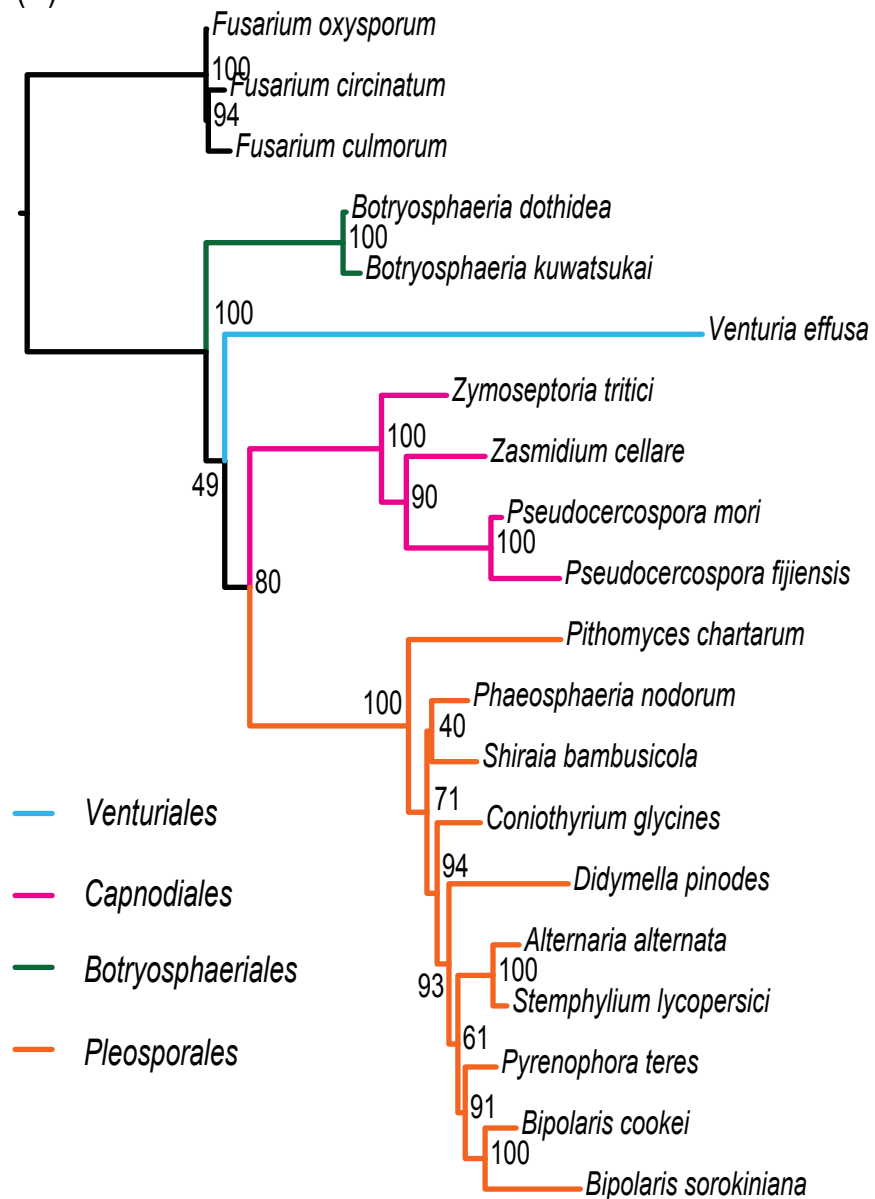

(B)

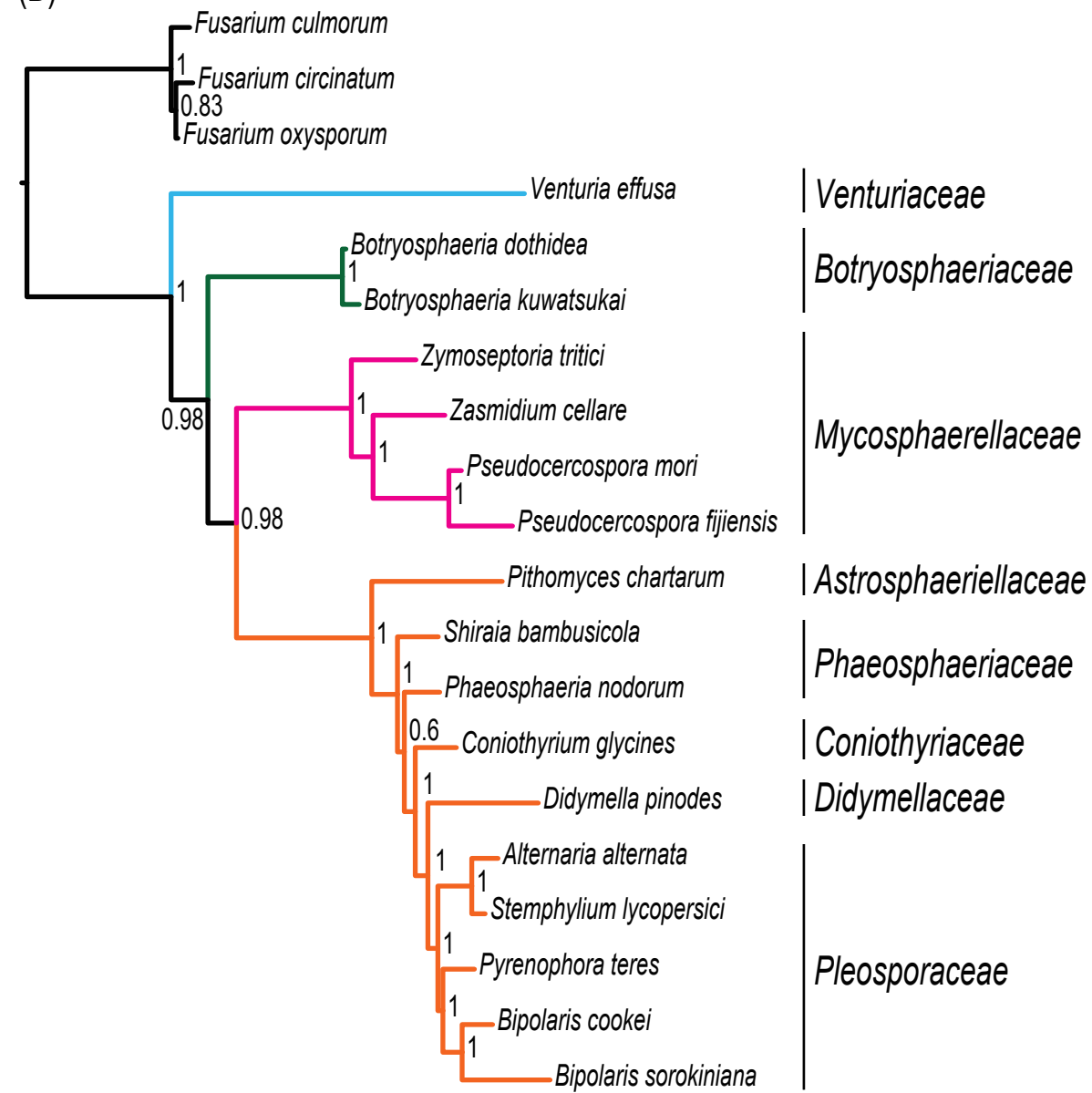

Supplement: Supplementary file 10 [file Image_3.PDF]

(A)

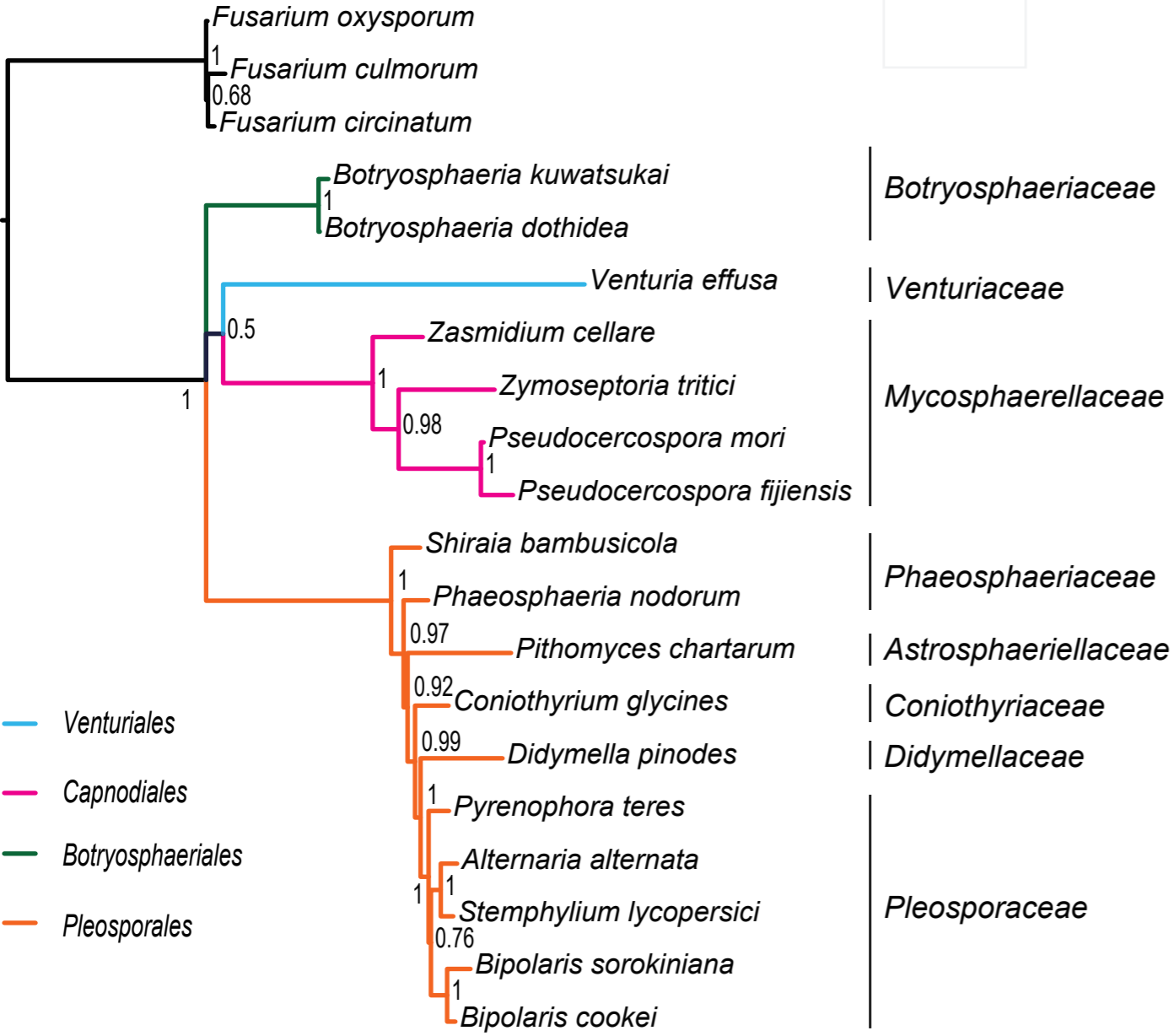

(B)

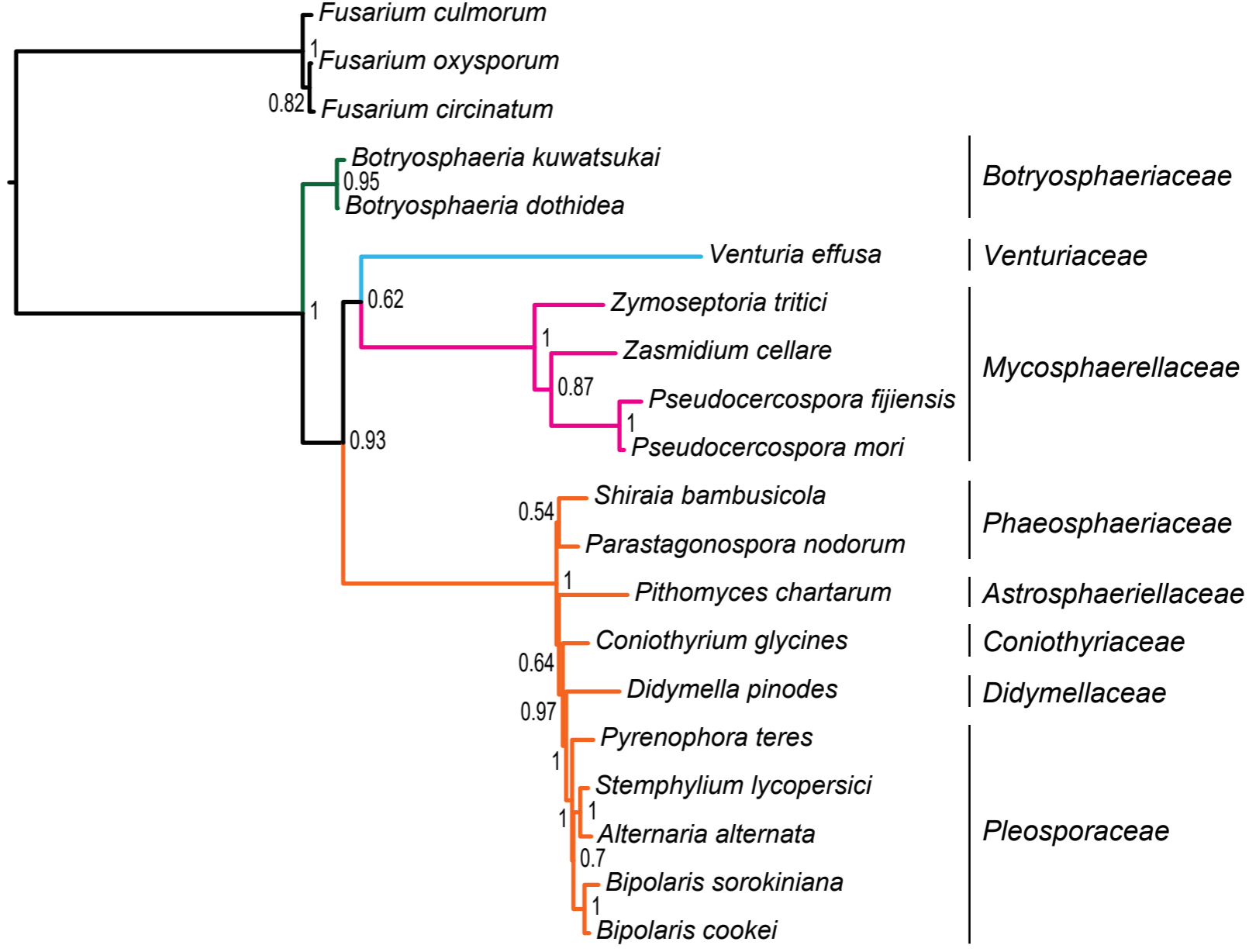

Supplement: Supplementary file 11 [file Image_4.PDF]

rrn

trn

Hypothesis

- a) Venturiales
- b) Capnodiales
- c) Botryosphaeriales
- d) Pleosporales

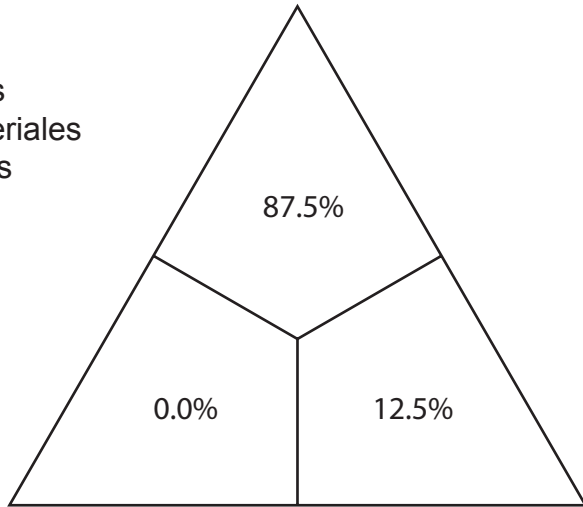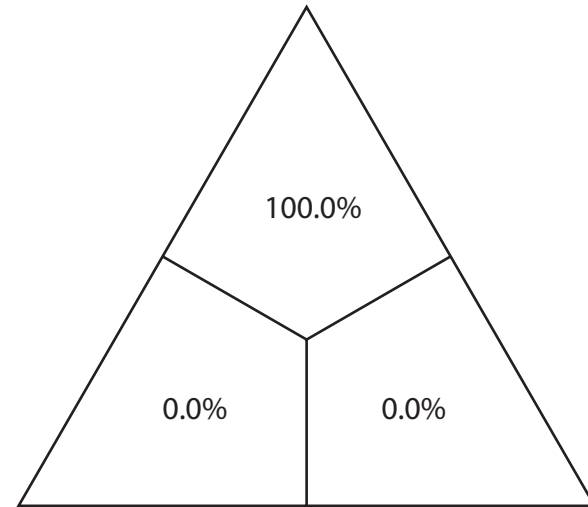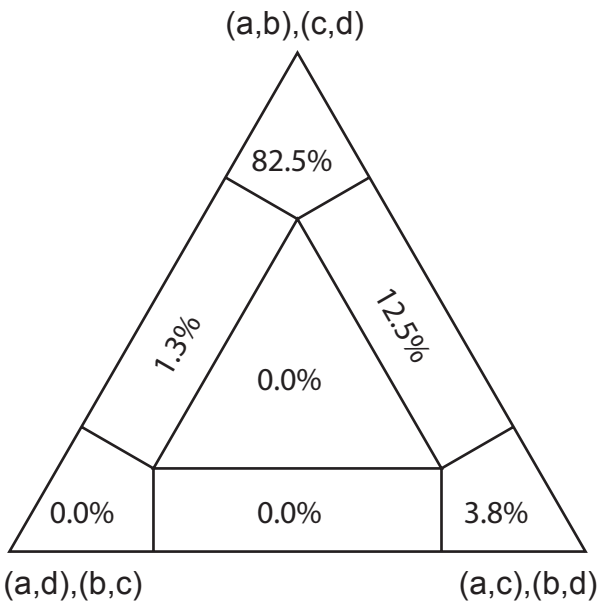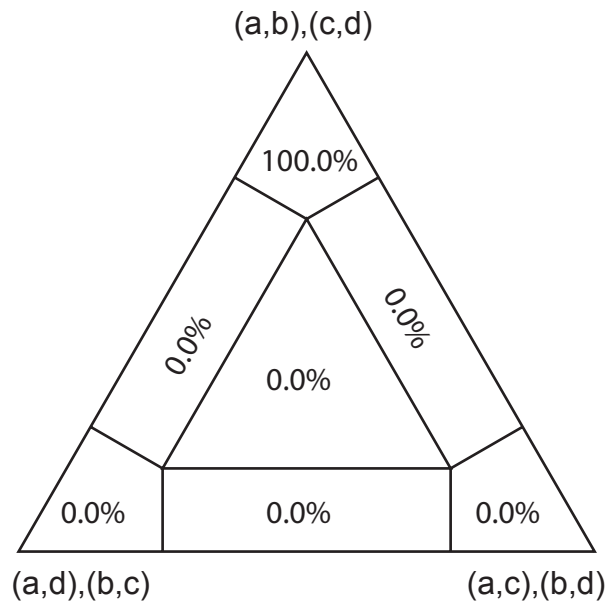

Supplement: Supplementary file 12 [file Image_5.PDF]
