# Supplementary material for: The Mitochondrial Genome of the Phytopathogenic Fungus Bipolaris sorokiniana and the Utility of Mitochondrial Genome to Infer Phylogeny of Dothideomycetes
Source: Front Microbiol. 2020 May 8;11:863. doi: 10.3389/fmicb.2020.00863 (PMC7225605; doi:10.3389/fmicb.2020.00863)
Supplement: Supplementary file 13 [file Image_6.PDF]

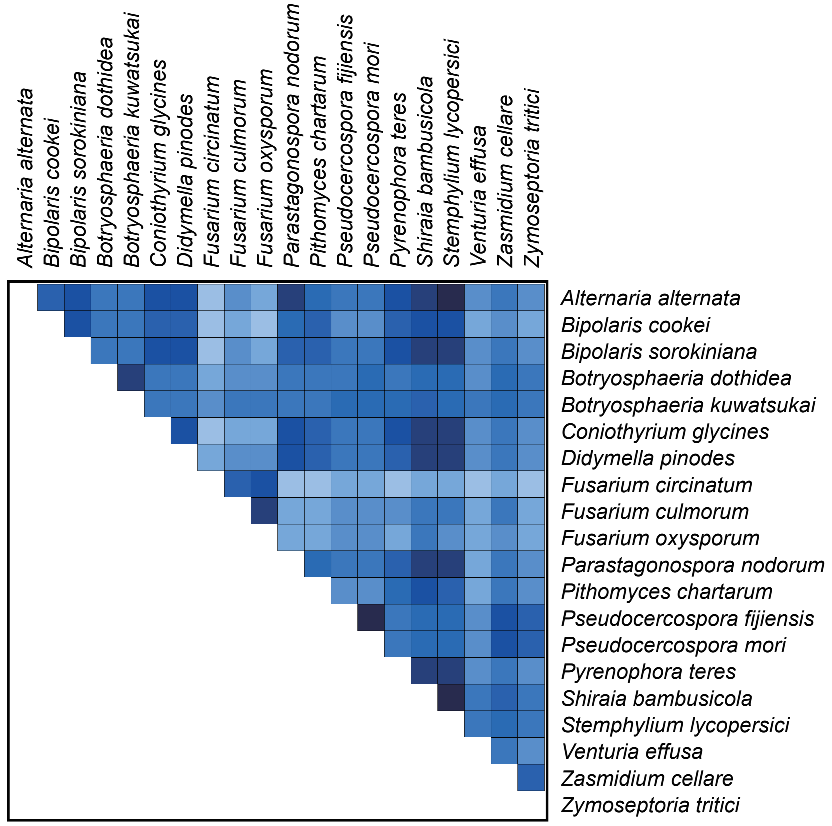


PCG-rrn(14,612 nucleotides)

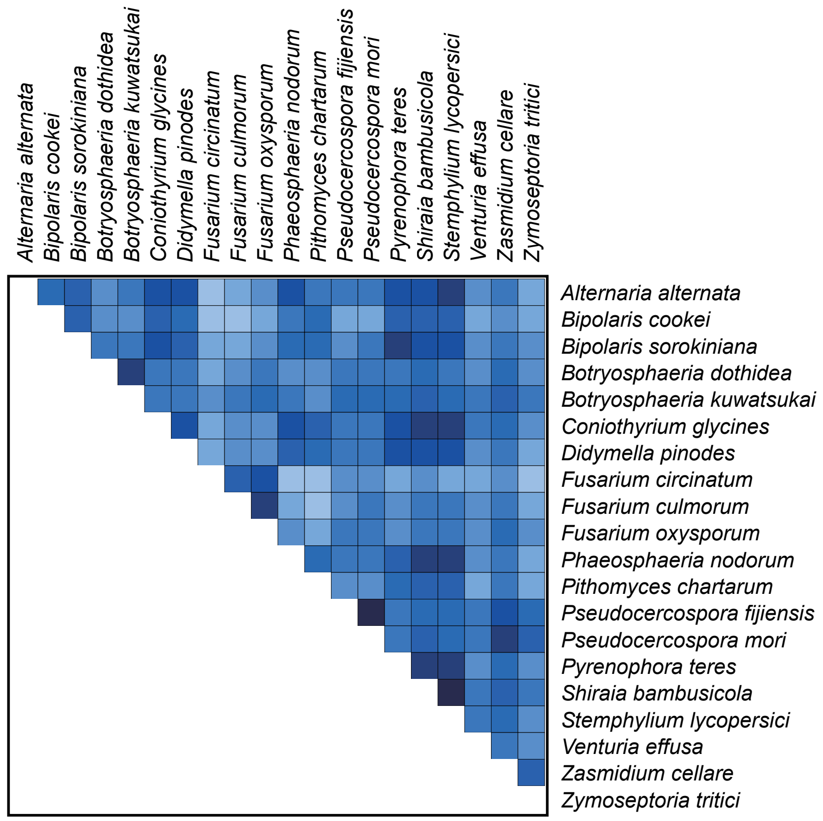


PCG_nt (10,561 nucleotides)

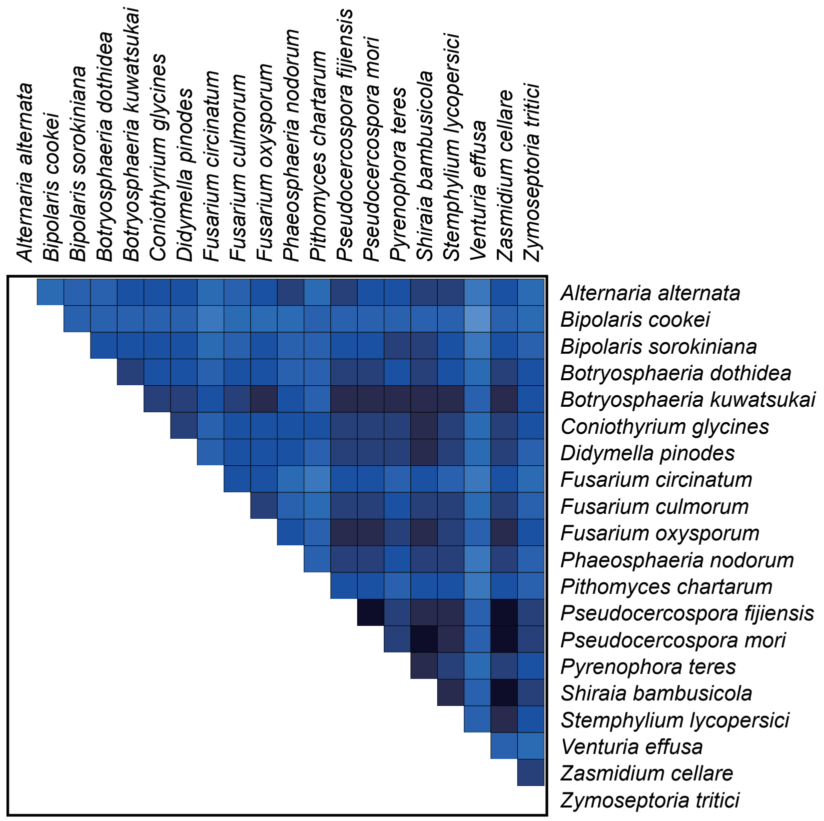


PCG_aa (3,364 amino acids)

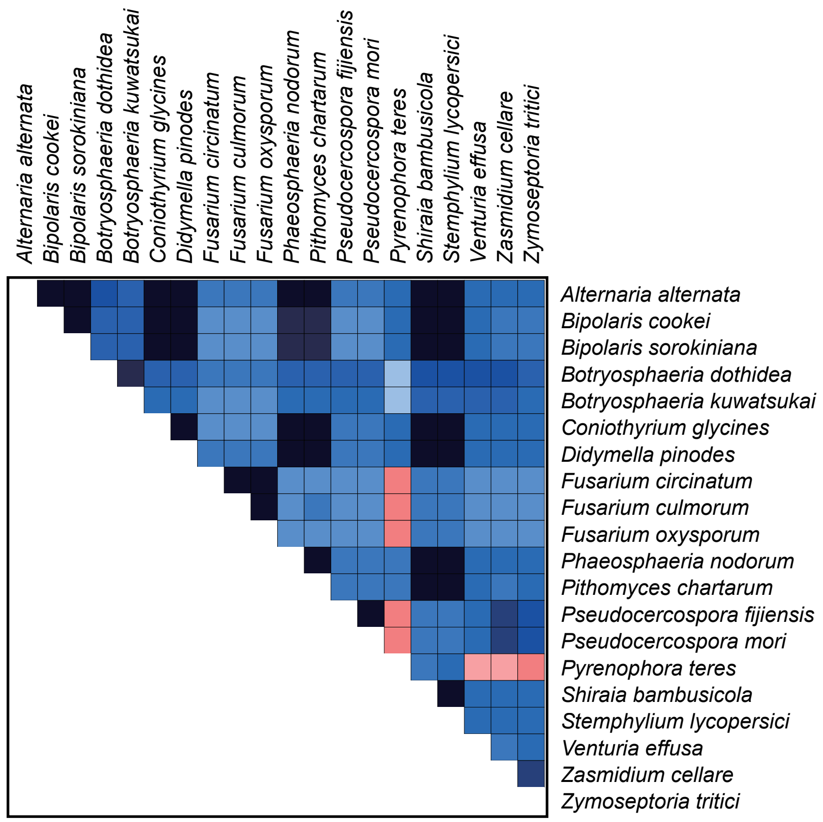


rrnS (1,390 nucleotides)

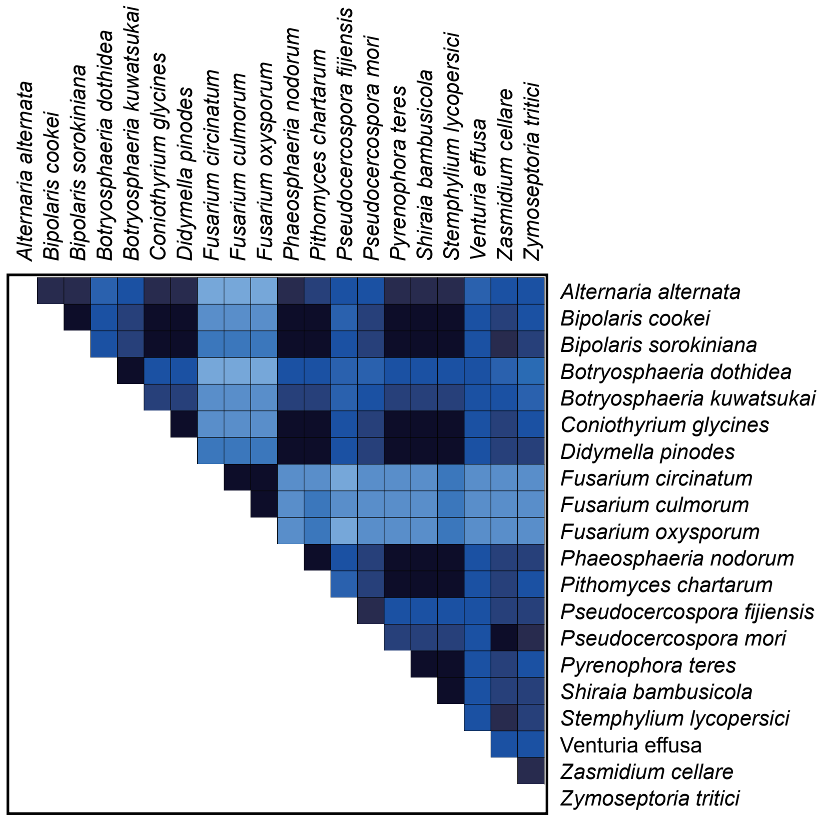


trn (1,637 nucleotides)
